# A New Human 3D-Liver Model Unravels the Role of Galectins in Liver Infection by the Parasite *Entamoeba histolytica*

**DOI:** 10.1371/journal.ppat.1004381

**Published:** 2014-09-11

**Authors:** Debora B. Petropolis, Daniela M. Faust, Gagan Deep Jhingan, Nancy Guillen

**Affiliations:** 1 Institut Pasteur, Cell Biology and Infection Department, Cell Biology of Parasitism Unit, Paris, France; 2 INSERM U786, Paris, France; 3 National Institute of Immunology, Signal Transduction Lab-1 Department, Aruna Asaf Ali Marg, New Delhi, India; University of Virginia Health System, United States of America

## Abstract

Investigations of human parasitic diseases depend on the availability of appropriate *in vivo* animal models and *ex vivo* experimental systems, and are particularly difficult for pathogens whose exclusive natural hosts are humans, such as *Entamoeba histolytica*, the protozoan parasite responsible for amoebiasis. This common infectious human disease affects the intestine and liver. In the liver sinusoids *E. histolytica* crosses the endothelium and penetrates into the parenchyma, with the concomitant initiation of inflammatory foci and subsequent abscess formation. Studying factors responsible for human liver infection is hampered by the complexity of the hepatic environment and by the restrictions inherent to the use of human samples. Therefore, we built a human 3D-liver *in vitro* model composed of cultured liver sinusoidal endothelial cells and hepatocytes in a 3D collagen-I matrix sandwich. We determined the presence of important hepatic markers and demonstrated that the cell layers function as a biological barrier. *E. histolytica* invasion was assessed using wild-type strains and amoebae with altered virulence or different adhesive properties. We showed for the first time the dependence of endothelium crossing upon amoebic Gal/GalNAc lectin. The 3D-liver model enabled the molecular analysis of human cell responses, suggesting for the first time a crucial role of human galectins in parasite adhesion to the endothelial cells, which was confirmed by siRNA knockdown of galectin-1. Levels of several pro-inflammatory cytokines, including galectin-1 and -3, were highly increased upon contact of *E. histolytica* with the 3D-liver model. The presence of galectin-1 and -3 in the extracellular medium stimulated pro-inflammatory cytokine release, suggesting a further role for human galectins in the onset of the hepatic inflammatory response. These new findings are relevant for a better understanding of human liver infection by *E. histolytica*.

## Author Summary

The study of liver infection is based on animal models, but the animal physiology does not always reflect the reality of the human host. This is particularly true for pathogens whose exclusive natural hosts are humans, such as *Entamoeba histolytica*, the protozoan parasite responsible for amoebiasis. Here, we constructed an experimental human 3D-liver model able to reproduce the first steps of amoebic hepatic infection (barrier crossing, tissue migration and pro-inflammatory reaction). Using this 3D-liver model we were able to decipher the first stages of hepatic invasion by *E. histolytica* and to unravel the role played by galectin-1 and galectin-3 during amoebic hepatic adhesion and pro-inflammatory reaction. Moreover, the model enables analysis usually not possible with *in vivo* samples, such as the quantification of pro-inflammatory cytokines released inside the tissue microenvironment. Our 3D-liver model has the potential to bridge the gap between animal models and the reality of the human host for the study of amoebic infection and other infectious diseases of the liver.

## Introduction

The protozoan parasite *Entamoeba histolytica* is the etiological agent of human amoebiasis. The parasite has a simple life cycle alternating the contaminating cyst and the vegetative trophozoite form. Infection occurs upon uptake of cysts with contaminated water or food and their differentiation into trophozoites, which colonize the intestine. Amoebae may breach the intestinal barrier and disseminate through the portal vein system, mainly to the liver (approximately 1% of the carriers). Hepatic amoebiasis is characterized by the induction of an inflammatory host response, the invasion of the liver parenchyma and the subsequent formation of abscesses, and leads to 70,000 deaths per year [1]. To study hepatic amoebiasis experimentally, we previously used hamsters (*Mesocricetus auratus)* since these are highly susceptible to *E. histolytica* infection and develop amoebic liver abscesses in a few days [2]. With this animal model we have shown that invasion of the liver parenchyma depends on amoebic adhesion to host cells through the activity of the galactose- or N-acetyl-galactosamine-inhibitable (Gal/GalNAc) lectin, the main amoebic adhesion [3]. For instance, trophozoites deficient in signalling through the Gal/GalNAc lectin (HGL-2 strain) form small foci close to the endothelium which do not progress to liver abscesses [4]. We also observed endothelial cell apoptosis in the vicinity of wild-type trophozoites as shortly as 1 h after infection, whereas with HGL-2 trophozoites cell death was delayed by almost 24 h [5]. However, the step in which HGL-2 trophozoites are blocked is currently not known.

Liver sinusoidal endothelial cells (LSEC) and hepatocytes are the major cellular components of the liver, accounting for around 80% of the liver mass [6], and LSEC constitute the first hepatic barrier during liver invasion. Little is known about the transmigration of *E. histolytica* through the hepatic endothelium, and the molecules required for interaction with LSEC. We have previously shown that cells of a human LSEC line respond in a localized manner to the presence of *E. histolytica* with changes in the integrin-mediated adhesion signalling, retraction, loss of their cytoskeleton organization and focal adhesion complexes [7]. These alterations may be relevant for the disease, by facilitating the endothelial barrier crossing or altering the immuno-modulatory function of LSEC. Further studies aiming to determine the mechanisms of amoebic adhesion to LSEC and endothelium crossing have been limited by the simplicity of two-dimensional (2D) cell culture systems and the difficulty to molecularly handle the high complexity of animal models. Moreover, animal data should be extrapolated with caution to the human disease since humans being the exclusive natural hosts for *E. histolytica* and rodents do not reproduce human liver physiology and immunology, in particular regarding human inflammatory diseases [8]. The development of a human 3D-liver model, consisting of cells grown in 3D-scaffolds and bridging the gap between cell cultures and tissues, would provide a new alternative for the study of human hepatic amoebiasis. While in the cell biology field the utility and advantages of *in vitro* tissue-like models are recognized, for infectious disease studies they have been used only rarely [9] [10] [11]. Tissue-like models allow the use of primary or immortalized human cells, the control of the non-cellular components of the microenvironment and analysis by advanced imaging techniques [12] [13]. Major advantages of this approach are the reduction of the complexity to a controlled but still physiologically relevant level and the possibility to define the roles of individual components at the molecular level.

The major objective of the present work was to analyse the early stages of hepatic sinusoid invasion by *E. histolytica*, in a human and physiologically relevant system. We intended to examine the mechanisms of *E. histolytica* adhesion to and crossing of the endothelial hepatic barrier, migration to and interaction with hepatocytes, and the induction of the immune response. To accomplish this objective we elaborated a human 3D-liver model mimicking basic features of the hepatic sinusoid environment. *In vivo*, LSEC form monolayers devoid of a basement membrane that are separated from hepatocytes by the Disse's space containing loosely organized extracellular matrix (ECM) components. Our 3D-liver model is thus composed of an LSEC layer co-cultivated on top of a hepatocyte layer embedded in a 3D collagen-I (COL-I) matrix. We chose to use cells from established cell lines, since primary cell cultures rapidly lose their hepatic phenotype [14] [15] and show higher phenotypic variability. The LSEC line used was established from non-tumour liver endothelial cell primary cultures, and cells maintain the expression of typical markers [16]. Huh-7 cells, though hepatoma-derived, are widely used for their phenotypic resemblance to differentiated hepatocytes [17] [18].

By analysing several physiological aspects such as sinusoid architecture, matrix porosity and barrier permeability, the presence of cell-cell and cell-ECM adhesion molecules, the expression of hepatic markers, as well as the secretion of soluble molecules, we demonstrated the value of the 3D-liver model for the study of hepatic invasion by *E. histolytica.* Two-photon microscopy was used to monitor interactions of *E. histolytica* with the 3D-liver model components. We investigated the organization of the LSEC and hepatocyte layers, as well as trophozoite cytoadhesion and matrix crossing, migration and cytotoxic effects. The data showed that LSEC efficiently function as an endothelial barrier for the parasite. Trophozoites impaired in Gal/GalNAc lectin (the major adhesin) function showed reduced ability to cross the LSEC barrier, indicating an important role for this protein in the early steps of liver invasion.

The 3D-liver model made possible long-term incubations with *E. histolytica* under serum-free conditions permitting to determine the secretome (proteome of soluble factors) composition and to identify new factors released upon amoebic interaction with the human cells. The analysis identified for the first time galectin family members participating in amoebic infection. Galectin has been reported to play a role during the innate immune response to several microbial infections [19] [20]. ELISA confirmed the presence of galectins and further indicated that the hepatic cells respond to amoebic interactions by a temporally and spatially organized pro-inflammatory reaction (IL-1β, IL-6, IL-8, TGF-β1). Cell adhesion and binding assays revealed the participation of galectin-1 and -3 in amoebic adhesion to cells. The role of galectins was confirmed by the striking reduction of amoeba adhesion to siRNA-treated LSEC exhibiting reduced galectin-1 levels. The data reveal a dual role of human galectin-1 and -3 and describe, for the first time, the participation of human galectins in hepatic infection with *E. histolytica*.

Taken together, the 3D-liver model we established we conclude on the combined action of amoebic Gal/GalNAc lectin and human galectins during the early steps of hepatic amoebiasis.

## Results

### A human 3D-liver model to study hepatic invasion by *E. histolytica*

**Figure 1:**
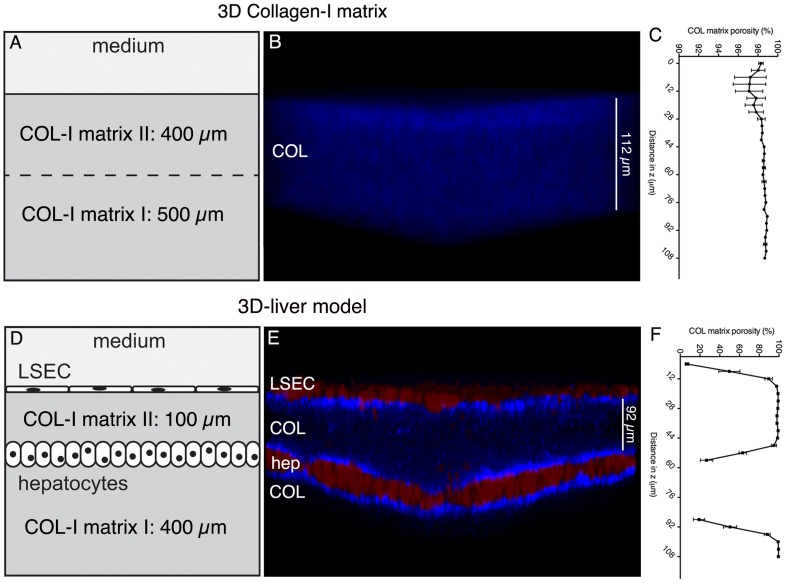
The human 3D-liver model. The 3D-liver model consists of co-cultured Huh-7 (hep) and LSEC layers and a COL-I matrix sandwich as mechanical ECM support for the cells, reproduces the layered structure of hepatic sinusoids in a 3D environment, at an *in vitro* micro-scale. (A–C) COL-I matrix without cells, and (D–F) 3D-liver model after 3d of culture. (B, E) Transversal view of 3D reconstructed images (ICY software). Red cell tracker-labelled hepatic cells visualized by two-photon microscopy and COL-I fibres (in blue) by SHG signals. Note that only the top parts of the images (around 130 µm) are represented. (C, F) COL-I matrix porosity at different z-stack positions without (C) or with (F) hepatic cells.

The human 3D-liver model we established is composed of a monolayer of Huh-7 hepatocytic cells embedded in a 3D COL-I matrix and an LSEC monolayer plated on top of the matrix facing the medium (Figure 1).

The 3D-liver model architecture organization was evaluated by two-photon microscopy and SHG visualization of the hepatic cells layers and matrix structure (Figure 1). The matrix structure in the absence and in the presence of the hepatic cells was compared demonstrating that without cells (Figure 1A–B) the COL-I fibres were homogeneously distributed throughout the matrix, which was at least 900 µm high. In the presence of the cells (Figure 1D–E), the same matrix layer had a mean height of only around 500 µm and its structure changed to a heterogeneous distribution of the COL-I fibres. The SHG signals of the fibres were more intense and the matrix porosity (Figure 1C and F) was significantly smaller in the vicinity of LSEC and hepatocytes, demonstrating that the hepatic cells remodel the matrix architecture of their 3D environment.

**Figure 2:**
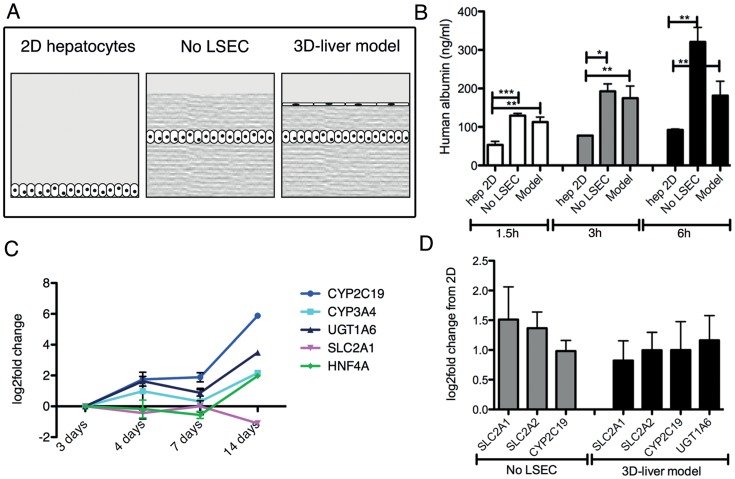
Expression of hepatocyte functions in the human 3D-liver model. (A) Schematic representation of the different set-ups used for the analyses. Standard 2D Huh-7 monocultures (2D hepatocytes), the COL-I sandwich with a Huh-7 monolayer (no LSEC) and the 3D-liver model with a Huh-7 and LSEC layer. (B) Human albumin secretion into the medium on top of the cultures measured by ELISA. Cultures were used 3d after seeding. Albumin secretion into fresh serum-free medium was determined after 1.5–6 h incubation. (C, D) Expression of hepatocyte markers by Q-RT-PCR analysis (see Figure S3 for complete datasets). (C) Amount of transcripts expressed in the 3D-liver model monitored over time of culture (3–14d). Changes in transcript levels were expressed as log2-fold changes in comparison to levels determined for the 3d cultures (set to 0). The graph represents the 5 markers whose expression was maintained over time. (D) Transcript levels in 3d cultures represented as log2-fold changes over levels in 2D Huh-7 cultures (set to 0). The graph shows the functions for which the levels were significantly increased. Note that for the 3D-liver model, normalization of Q-PCR data with GAPDH (expressed by both hepatocytes and LSEC) leads to an underestimation of the transcript level for the hepatocyte-specific markers.

To quantify the ability of *E. histolytica* to cross the LSEC layer and to determine the rate of migration in the 3D matrix towards the hepatocytes we used a 100 µm mean distance between LSEC and hepatocytes enabling the distinction of the compartments (Figure 1). This matrix height takes into account the size of trophozoites ranging from 25 to 50 µm and their high motility (10 µm/sec). The distance between LSEC and hepatocytes diminished progressively over culture time and the most reproducible 100 µm mean distance was obtained upon three days of culture. Furthermore, to decipher the individual contributions of LSEC and hepatocytes to the response to amoebae we compared the 3D-liver model with setups lacking either the LSEC or the hepatocyte layer (schematically represented in Figure 2A).

### Characterization of physiological markers of the 3D-liver model

Several characteristics of the 3D-liver model were analysed to determine its physiological relevance. Cell morphology, growth and the presence of cell-cell and cell-ECM adhesion molecules were monitored over time. By immunofluorescence microscopy, LSEC and hepatocytes were positive for ICAM-1 and integrin-β1, and hepatocytes showed a strong E-cadherin surface labelling (Figure S1). To evaluate LSEC barrier function, monolayer permeability was determined through the ability of 1 µm diameter fluorescent microspheres to penetrate into the compartments of the 3D-liver model (Figure S2). After 3 h of incubation, only 0.14% (mean number) of the beads added on top of the LSEC layer were found in the matrix beneath. In the absence of LSEC, the proportion of beads inside the matrix was comparable to the matrix without cells (around 80%) and the beads stopped at the position of the hepatocyte layer. Data show that both the LSEC and hepatocyte layer of the 3D-liver model are efficient barriers for 1 µm particles.

Albumin secretion and the expression of transcripts encoding several hepatocyte-specific functions were compared between the 3D-liver model (with or without LSEC) and Huh-7 standard 2D monocultures (Figure 2). Albumin release was significantly higher in the 3D-liver model than in 2D cultures in all time points analysed (Figure 2B). The hepatic markers we chose are either involved in drug metabolism as cytochromes P-450 (CYP2C19, CYP3A4) and UDP glucuronosyltransferase-1A6 (UGT1A6) or belong to the solute carrier transporter family (SLC2A1, SLC2A2). In addition, we tested expression of hepatocyte nuclear factor-α (HNF4A), a key transcription factor for many hepatic genes. The expression of most of those genes was maintained or increased between the 3- to 14d-period tested (Figure 2C and S3). Moreover, transcript levels for SLS2A1, SLC2A2, CYP2C19 and UGT1A6 were higher in the 3D-liver model than in the 2D culture at the 3d time-point (Figure 2D and S3).

Together the results demonstrate that the 3D-liver model retains hepatic barrier performance and hepatocyte functionality in a more physiological environment than standard 2D cell cultures. For the experiments described below we used the 3D-liver model 3d after having started its preparation.

### Protein release by hepatic cells in the 3D-liver model

The 3D-liver model offers the possibility to characterize the molecules released by the cells into the culture medium on its top, since in this model LSEC have lost the strong serum dependence observed in conventional cell culture conditions, i.e. they are viable, morphologically normal and express adhesion markers ICAM-1 and integrin-β1 for at least 12 h without serum (data not shown). The compounds released were identified after 3 h in fresh serum-free medium, using liquid chromatography–mass spectrometry (LC-MS/MS) analysis (Table S1). From the 64 human-specific proteins identified (Table S2), the 45 proteins known for being released were grouped according to their main functions (Table 1). Several components and regulators of the blood coagulation (9 proteins), the complement cascade and the innate immune response (7 proteins) were detected. Within the group of 11 plasma transporters, all exclusively or mainly synthesized in hepatic cells, well-known hepatic markers [14] were present, such as serum albumin, α-fetoprotein, apolipoproteins A-I, A-II, B-100, and E. In addition to COL-I, the ECM components fibronectin, nidogen-1, and fibrinogens were found, as well as several adhesion molecules. These results reveal that a variety of hepatic functions are expressed and that the hepatic cells are capable to enrich their micro-environment in the 3D-liver model.

**Table 1:** Human proteins released into the medium in top of the 3D-liver model.

Accession	Name	Short Name	Coverage	Proteins groups	Unique Peptides	Peptides	PSMS	MW [kDa]
**ECM/Cell adhesion proteins**								
P02452	Collagen alpha-1(I) chain		4.17	1	3	3	16	138.9
P02671	Fibrinogen alpha chain		7.74	1	4	4	13	94.9
P02675	Fibrinogen beta chain		28.92	1	8	8	17	55.9
P02679	Fibrinogen gamma chain		22.30	1	6	6	17	51.5
**P02751**	**Fibronectin**	FN	11.06	1	13	13	28	262.5
P04004	Vitronectin	VN	15.27	1	4	4	7	54.3
P14543	Nidogen-1	NID-1	2.33	1	1	1	2	136.3
P19823	Inter-alpha trypsin inhibitor heavy chain H2	ITI heavy chain H2	17.86	1	10	10	24	106.4
Q15582	Transforming growth factor-beta-induced protein ig-h3	Beta ig-h3	7.61	1	2	2	5	74.6
**P05546**	**Heparin cofactor 2**	HC-II	7.62	1	3	3	4	57.0
**Blood coagulation regulation**								
P00749	Urokinase-type plasminogen activator	Uplasminogen activator	2.32	1	1	1	1	48.5
P01008	Antithrombin-III	ATIII	15.30	1	5	5	14	52.6
P01042	Kininogen-1	HMWK	6.68	1	3	3	4	71.9
P02671	Fibrinogen alpha chain		7.74	1	4	4	13	94.9
P02675	Fibrinogen beta chain		28.92	1	8	8	17	55.9
P02679	Fibrinogen gamma chain		22.30	1	6	6	17	51.5
**P05121**	**Plasminogen activator inhibitor 1**	PAI	4.98	1	1	1	2	45.0
**P05546**	**Heparin cofactor 2**	HC-II	7.62	1	3	3	4	57.0
P05452	Tetranectin	TN	17.33	1	2	2	3	22.5
**Plasma transporters**								
**P02647**	**Apolipoprotein A-I**	Apo-AI	49.81	1	12	12	49	30.8
**P02649**	**Apolipoprotein E**	Apo-E	11.67	1	2	2	5	36.1
**P02652**	**Apolipoprotein A-II**	Apo-AII	42.00	1	2	2	4	11.2
**P02765**	**Alpha-2-HS-glycoprotein**		34.33	1	6	6	23	39.3
**P02768**	**Serum albumin**		58.29	1	32	32	555	69.3
**P02771**	**Alpha-fetoprotein**		73.73	1	30	30	429	68.6
**P02774**	**Vitamin D-binding protein**	DBP	34.81	1	11	11	77	52.9
**P00738**	**Haptoglobin**		16.01	1	2	2	3	45.2
**P02787**	**Serotransferrin**	Transferrin	11.89	1	6	6	20	77.0
**P02790**	**Hemopexin**		3.46	1	1	1	1	51.6
**P04114**	**Apolipoprotein B-100**	Apo B-100	6.82	1	19	19	35	515.3
**Complement cascade/Innate immune response**								
P01024	Complement C3	ASP	18.88	1	20	20	61	187.0
P01031	Complement C5							
**P02763**	**Alpha-1-acid glycoprotein 1**	AGP 1	4.48	2	1	1	1	23.5
P05155	Plasma protease C1 inhibitor	C1 Inh	4.00	1	2	2	2	55.1
P05156	Complement factor I		3.09	1	1	1	2	65.7
**P08603**	**Complement factor H**		8.20	1	6	6	6	139.0
**P02760**	**Protein AMBP**	Protein HC	21.31	1	6	6	19	39.0
**Blood/Vascular homeostasis**								
**P01019**	**Angiotensinogen**	Ang I	34.43	1	8	8	25	53.1
**P08697**	**Alpha-2-antiplasmin**	Alpha-2-AP	9.78	1	3	3	7	54.5
P10909	Clusterin	Apo-J	16.70	1	6	6	7	52.5
P36955	Pigment epithelium-derived factor	PEDF	29.90	1	6	6	18	46.3
**Q8NBP7**	**Proprotein convertase subtilisin/kexin type 9**	NARC-1	3.18	1	1	1	2	74.2
**Enzymes**								
O00391	Sulfhydryl oxidase 1	hQSOX	2.68	1	1	1	1	82.5
Q5FYB0	Arylsulfatase J	ASJ	5.68	1	1	1	1	67.2

Proteome data after BLAST search with the *H. sapiens* UniProt database (samples from 4 biological replicates) were cleaned from possible bovine serum contaminants by eliminating any peptides or proteins shared with bovine species. A total of 64 proteins having at least one human-specific peptide was identified (see Table S1 and S2). A literature search and the information contained in the UniProtKB/Swiss-Prot database identified 45 proteins known for being released or secreted. Proteins were grouped according to their main function given in the UniProtKB/Swiss-Prot database and organized by the alphabetical order of the database accession numbers. Bold-labelled entries correspond to those proteins for which hepatic cells are known to be the exclusive or a main site of synthesis. The numbers given are average from all samples. PSMS is peptide spectrum matches, MW molecular weight.

### Invasion of the human 3D-liver model by virulent and virulence-attenuated *E. histolytica*

**Figure 3:**
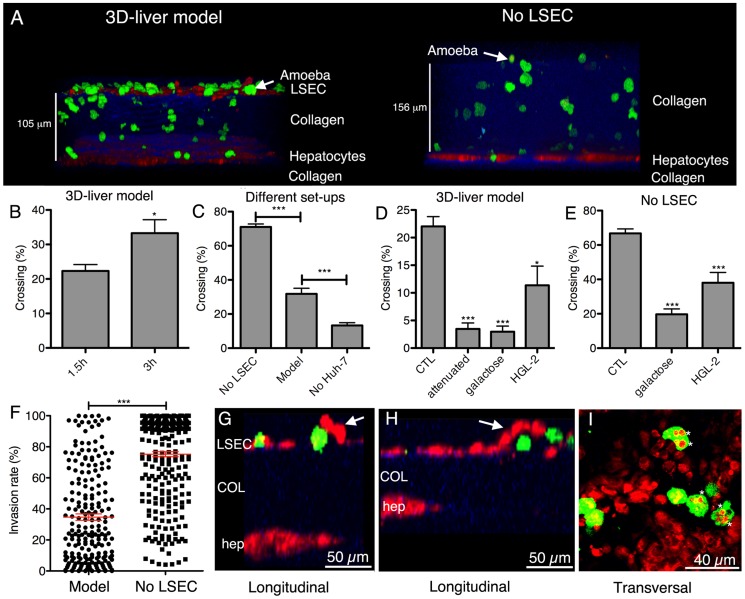
*E. histolytica* crossing and invasion. (A) Transversal view of 3D reconstructed two-photon microscopy and SHG images (ICY software) of the 3D-liver model or the setup without LSEC (No LSEC) after 3 h of interaction with virulent amoebae. Hepatic cells (red), amoebae (green) and COL-I (blue). (B–E) Quantification of the amoebae crossing the 3D-liver model or the different setups, expressed as the percentage of amoebae crossing the LSEC layer or penetrating the COL-I matrix, from the total number of amoebae in the field. (B) Incubation of the 3D-liver model with virulent amoebae for 1.5 h and 3 h. (C) Incubation of the different setups with virulent amoebae for 3 h. (D–E) Incubations with different amoebae strains, and with glucose or galactose of (D) the 3D-liver model for 1.5 h, or (E) the setup without LSEC for 3 h. Values obtained for incubations with virulent trophozoites in the absence or the presence of glucose, or with the HGL-2 control trophozoites transfected with the vector not containing Gal/GalNAc lectin sequences, were not significantly different and are thus represented together as control (CTL). (F) Amoebic invasion rate at 3 h, in the presence (Model) or in the absence (No LSEC) of LSEC, with 0% invasion set at the LSEC and 100% at the hepatocyte layer z-position. Red bars indicate the means. Graphs with standard deviations and statistical evaluation by One way ANOVA, p<0.01*, <0.0001*** for n = 50 of minimal 3 independent experiments. (G–I) Amoebic interactions (3 h) with the LSEC layer of the 3D-liver model. (G–H) Arrows point to LSEC detaching in the vicinity of amoebae (I). Asterisks mark vesicular structures localized inside trophozoites and labelled with cell tracker used for hepatic cell staining.

The 3D-liver model was used to study initial steps of liver invasion by *E. histolytica*. Virulent (i.e. inducing amoebic liver abscesses in the hamster) trophozoites were added to the medium on top of the 3D-liver model (Figure 3A). The majority of the amoebae adhered to the LSEC layer. For at least 6 h, all trophozoites localized inside the 3D-liver model were migrating and their mobility indicates that cells were likely alive. Approximately 20% to 30% of the amoebae have crossed the LSEC barrier (Figure 3B) after incubation for 1.5 h or 3 h, respectively. In the absence of hepatocytes, amoebic invasion was significantly lower (Figure 3C), suggesting the existence of attractant molecules secreted by hepatocytes or a difference in a potentially mechanical effect of the remodelled matrix. In the absence of the LSEC layer (Figure 3A) more than 60% of the amoebae crossed the matrix after 3 h (Figure 3C) and their migration towards the hepatocytes was significantly increased resulting in a different invasion rate profile (Figure 3F). Thus, the LSEC monolayer behaves as an efficient barrier for trophozoite invasion. Virulence-attenuated trophozoites (unable to produce liver abscesses in the hamster) adhered to LSEC in the 3D-liver model as efficiently as virulent trophozoites. However, their capacity to invade is significantly diminished, with less than 5% of the trophozoites crossing the LSEC barrier after 1.5 h (Figure 3D) and no significant increase after 3 h of interaction (data not shown), showing their reduced ability to efficiently leap over the first barrier of liver infection, i.e. LSEC crossing.

### Changes in the hepatic cell monolayers upon interaction with virulent *E. histolytica* in the 3D-liver model

To characterize the morphological changes in the human cell monolayers upon interaction with *E. histolytica*, two-photon microscopy and SHG were used. During amoebic interaction with the LSEC, local detachment of individual cells from the COL-I matrix was frequently observed at sites of amoebae crossing the layer (Figure 3G and H) but areas of detachment did not obviously extend over time, suggesting the existence of a replacement or repair mechanism. Trophozoites containing vesicles labelled with the cell tracker used for the hepatic cells were frequently and specifically observed in the vicinity of the LSEC layer (Figure 3I). Amoebic engulfment of LSEC portions could either originate from the detachment and the subsequent uptake of portions of live LSEC by trogocytosis, [21], or from phagocytosis of apoptotic bodies or dead cell debris.

Trophozoites rapidly migrated through the matrix in the direction of the hepatocytes (150 µm in 1.5 h). The percentage of amoebae crossing the hepatocyte layer was low and phagocytic-like structures were not frequent in trophozoites having reached the hepatocytes. No detachment or formation of gaps was observed during amoebic interaction with the hepatocyte monolayer, even after 6 h of interaction. However, immunofluorescence experiments with antibodies against E-cadherin, a marker for epithelial tight junctions, revealed a clear reduction of the signal in the presence of *E. histolytica* (Figure S4). This reduction could reveal changes in cell-cell contacts that may ultimately increase the epithelial barrier permeability and facilitate the crossing of the hepatocyte barrier after longer incubation periods.

### Gal/GalNAc lectin-dependent *E. histolytica* interaction with hepatic cells in the 3D-liver model

Adhesion to mammalian cells is a prerequisite for the cytotoxic effects of *E. histolytica* and depends upon the amoebic Gal/GalNAc lectin [3]. To examine its role in the penetration of the 3D-liver model, the effect of galactose on trophozoite invasion was first analysed. Galactose almost completely abolished the ability of virulent trophozoites to cross the LSEC layer (Figure 3D), the proportion of amoebae being about five times lower than for the glucose control (CTL). Amoebic crossing of the COL-I matrix (tested in the setup without LSEC) was also significantly reduced in the presence of galactose (Figure 3E).

To further investigate the participation of Gal/GalNAc lectin, amoebic transfectants (HGL-2 strain) were used expressing a dominant-negative form of the lectin [4]. Compared to control transfectants (CTL), HGL-2 trophozoites presented a significant reduction in the LSEC (Figure 3D) and matrix crossing activity (Figure 3E). The less pronounced effects found with HGL-2 transfectants compared with galactose could be due to the fact that in HGL-2 trophozoites only the intracellular signalling of Gal/GalNAc lectin is blocked, while the extracellular function remains unchanged [5].

### Protein release by hepatic cells upon *E. histolytica* invasion of the 3D-liver model

Secretome analysis was performed to identify human proteins released during amoebic hepatic invasion (Table S1). Products released into the medium were determined after 3 h interaction with virulent *E. histolytica*. Within the 139 human-specific proteins found exclusively in response to amoebae (Table S2) many human cytoskeletal proteins were present that likely originate from dying cells having lost their plasma membrane integrity. Note that the induction of host cell death is a main feature of *E. histolytica* infection. We found 24 known released or surface-associated proteins (Table 2), comprising further components of the complement cascade (3 proteins) and the blood coagulation system (4 proteins), and 7 proteins involved in cell/cell or cell/ECM interactions. Among the 8 proteins with functions in antigen presentation and immune responses, macrophage migration inhibitory factor (pro-inflammatory cytokine MIF), galectin-1, and galectin-3 binding protein were present. Galectin-1 is a regulator of a variety of immune responses and inflammation in host–pathogen interactions. Interestingly, galectin-1 has not been described before in the context of amoebic liver infection.

**Table 2:** Human released or cell surface-associated proteins identified in the medium on top of the 3D-liver model during *E. histolytica* invasion (specific for the 3D-liver model with amoebae).

Function Category	Accession	Protein Name	Short Name	Coverage	Proteingroups	Unique Peptides	Peptides	PSM	MW [kDa]
IMMUNE RESPONSE	P14174	Macrophagemigration inhibitory factor	MIF	18.49	1	1	1	6	13.7
IMMUNE RESPONSE	P61769	Beta-2-microglobulin		18.84	1	4	4	9	38.3
BLOOD COAGULATION	**P02749**	**Beta-2-glycoprotein 1**	Apo-H	30.37	1	2	2	5	14.7
IMMUNE RESPONSE ADHESION (ECM/Cell-cell)	P09382	Galectin-1	Gal-1	2.22	1	1	1	2	65.3
ADHESION HOST DEFENSE	Q08380	Galectin-3-binding protein	MAC2BP	3.58	1	2	2	3	129.9
CELL SURFACE ADHESION (ECM/Cell-cell)	P35442	Thrombospondin-2		4.68	3	2	2	4	45.6
BLOOD COAGULATION IMMUNE RESPONSE	**P05154**	**Plasma serine protease inhibitor**	PAI-3	18.91	1	3	3	12	23.0
TRANSPORTER	P02753	Retinol-binding protein 4	PRBP	2.53	1	1	1	1	84.7
CELL SURFACE ADHESION (Cell-Cell)	Q9ULH4	Leucine-rich repeat and fibronectin type-III domain-containing protein 2		12.33	1	1	1	1	51.1
CELL SURFACE UNKNOWN FUNCTION	Q5SWX8	Protein odr-4 homolog	hODR-4	1.68	1	1	1	1	388.1
ADHESION (ECM)	**P78509**	**Reelin**		2.69	1	1	1	1	29.4
CELL SURFACE IMMUNE RESPONSE	P20036	HLA class II histocompatibility antigen. DP alpha 1 chain		12.38	1	1	1	1	11.7
UNKNOWN FUNCTION	P10599	Thioredoxin	Trx	1.49	2	1	1	1	192.7
COMPLEMENT	**P0C0L4**	**Complement C4-A**		17.81	2	4	4	5	35.9
BLOOD COAGULATION	P08758	Annexin A5	CBP-I	41.30	2	10	10	51	38.6
MEMBRANE BINDING	P07355	Annexin A2	PAP-IV	7.17	1	3	3	3	63.1
ANGIOGENIC FACTOR	P06744	Glucose-6-phosphate isomerase	GPI	2.02	1	1	1	1	38.2
ADHESION (ECM)	P02750	Leucine-rich alpha-2-glycoprotein	LRG	2.11	1	2	2	4	138.5
ADHESION (ECM)	P02461	Collagen alpha-1(III) chain		6.63	32	1	1	1	40.9
CELL SURFACE IMMUNE RESPONSE	P01893	Putative HLA class I histocompatibility antigen. alpha chain H		1.83	1	1	1	1	85.5
COMPLEMENT	P00751	Complement factor B	GBG	4.32	1	3	3	3	90.5
BLOOD COAGULATION ECM REMODELING IMMUNE RESPONSE	**P00747**	**Plasminogen**		2.55	1	1	1	1	80.1
COMPLEMENT	P00736	Complement C1r subcomponent		7.50	1	1	1	1	26.8
GROWTH FACTOR	P51858	Hepatoma-derived growth factor	HDGF	18.49	1	1	1	6	13.7

Proteome data after BLAST search with the *H. sapiens* UniProt database (samples from 9 biological replicates) and two-stage filtering (i.e. elimination of bovine matches and subtraction of proteins identified in the model without amoebae) identified 139 proteins having at least one human-specific peptide (see Table S1 and S2). Further analysis was performed to identify secreted and cell membrane-associated proteins performing a literature search and information contained in the UniProtKB/Swiss-Prot database. For the 24 proteins found, their main function (given by the UniProtKB/Swiss-Prot database) is indicated in the table. Database accession numbers are provided. The proteins were ranked according to the number of samples in which they were found (decreasing from top to bottom). Bold-labelled entries correspond to those proteins for which hepatic cells are known to be the exclusive or a main site of synthesis. The numbers given are average from all samples. PSMS is peptide spectrum matches, MW molecular weight.

### Growth factor and cytokine release upon interaction with *E. histolytica*

**Figure 4:**
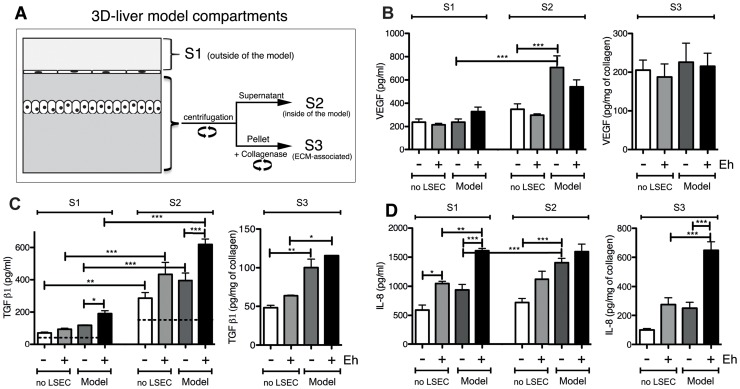
Human cytokine and growth factor profile in the absence or the presence of *E. histolytica*. (A) Preparation of the samples used for ELISA assays. (B–D) ELISA quantification in compartments S1, S2 and S3 of the 3D-liver model (Model) or the setup without LSEC (no LSEC), in the absence or the presence of *E. histolytica* (− or + Eh). (B) Amounts of VEGF at 6 h, (C) TGFβ1 at 3 h and (D) IL-8 at 6 h of incubation with fresh serum-free medium containing or not virulent *E. histolytica*. Graphs with standard deviation and statistical evaluation by One way ANOVA, p<0.01*, <0.001**, <0.0001*** for 3–5 independent experiments. As a control, cytokines/growth factors were quantified in the COL-I matrix without hepatic cells (detectable level mean values given as broken lines). n/d = not detected.

The identification of the immune-regulatory proteins MIF and galectin-1 in the secreted fraction suggests that cytokines may be released upon *E. histolytica* interaction with the 3D-liver model, which may not have been discovered in the secretome analysis due to their low abundance and/or degradation during the procedure. To increase the sensitivity of detection, we next performed ELISA for a selection of cytokines (IFNγ, IL-1β, IL-6, IL-8, TNFα, TGF-β1), growth factors (acidic FGF, HGF and VEGF) and in addition, galectin-1 and galectin-3. Analysis was carried out for the 3D-model without and with *E. histolytica*, and the setup without LSEC as control. Samples were prepared from 3 distinct compartments of the 3D-liver model (Figure 4A). The first corresponded to the medium on top (outside) of the 3D-liver model (supernatant S1, as used for the proteome approach), the second to the medium inside the 3D-liver model (supernatant S2), and the third to the matrix- and cell-associated molecules liberated after collagenase treatment of the non-soluble fraction (S3).

**Figure 5:**
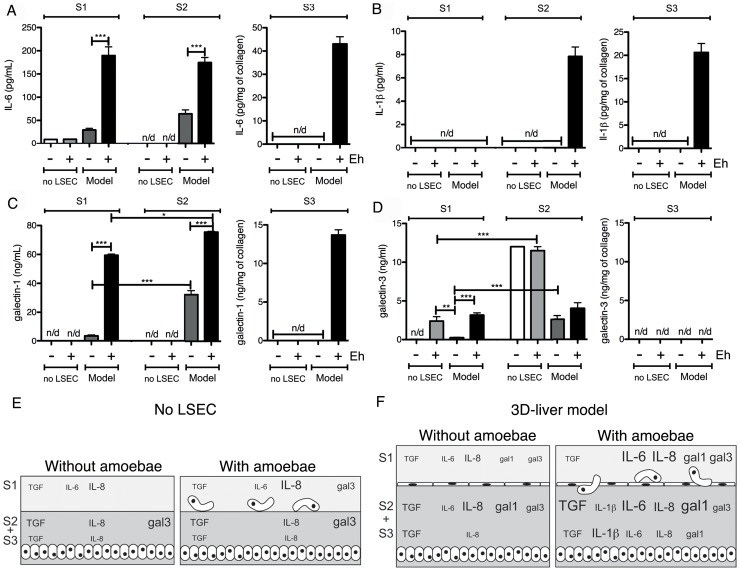
Human cytokine and growth factor profile in the absence or the presence of *E. histolytica*. (A–D) ELISA quantification in compartments S1, S2 and S3 of the 3D-liver model (Model) or the setup without LSEC (no LSEC), in the absence or the presence of *E. histolytica* (− or + Eh). Samples used for ELISA assays were prepared as for Figure 4. (A) Amounts of IL-6 at 3 h, (B) IL-1β, (C) galectin-1 and (D) galectin-3 at 6 h of incubation with fresh serum-free medium containing or not virulent *E. histolytica*. Graphs with standard deviation and statistical evaluation by One way ANOVA, p<0.01*, <0.001**, <0.0001*** for 3–5 independent experiments. None of the cytokines was detected in the COL-I matrix control without hepatic cells. n/d = not detected. (E, F) Summary of the results from Figure 4 and Figure 5, for the setup without LSEC (E) and the 3D-liver model (F), showing the complexity of the pro-inflammatory response induced by *E. histolytica* in the 3D-liver model.

FGF, HGF, IFNγ and TNFα were not found in any fraction tested. VEGF (Figure 4B) and TGF-β1 (Figure 4C) were readily detected in the three compartments of all samples, with different quantities. Without amoebae, IL-8 (Figure 4D) was, as expected, easily detected in all fractions, IL-6 (Figure 5A) only in low amounts in S1 and S2 of the 3D-liver model, whereas IL-1β (Figure 5B) was not found. Low galectin-1 (Figure 5C) and galectin-3 (Figure 5D) levels were present in the soluble fractions of the 3D-liver model.

**Figure 6:**
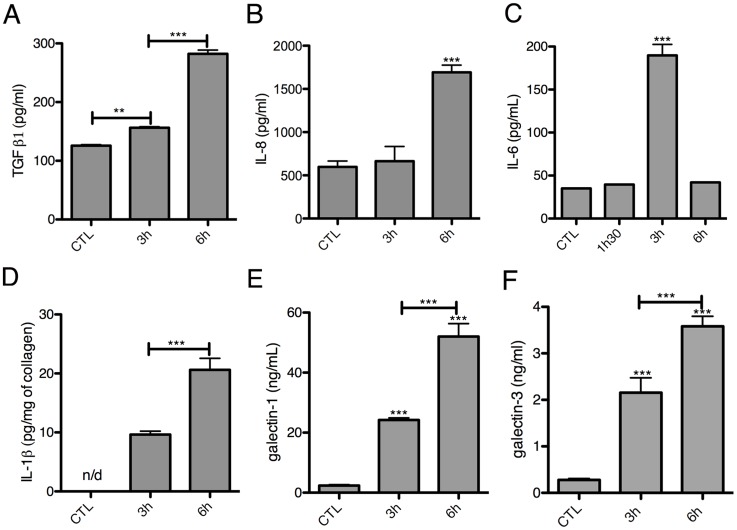
Kinetics of cytokine release in the 3D-liver model in response to *E. histolytica*. Quantification in fractions S1 (A–C, E–F) or S3 (D) after addition to the 3D-liver models of fresh serum-free medium containing virulent *E. histolytica*. Graphs with standard deviation and statistical evaluation by One way ANOVA, p<0.001**, <0.0001*** for minimal 3 independent experiments. CTL is incubation for 6 h without amoebae. n/d = not detected.

In the presence of amoebae, the VEGF release was unchanged (Figure 4B), but many significant modifications in the cytokine profiles occurred, concerning different compartments (Figure 5E–F) and showing distinct kinetics (Figure 6). The presence of amoebae significantly increased the amounts of TGF-β1 (Figure 4C) in the soluble fractions, IL-8 (Figure 4D) augmented in S1 and S3, and IL-6 (Figure 5A) and galectin-1 (Figure 5C) in all fractions of the 3D-liver model. Galectin-3 (Figure 5D) was only increased in S1. Interestingly, IL1-β (Figure 5B), undetectable in the absence of amoebae, was revealed inside the 3D-liver model (Figure S2 and S3) after 3 h and 6 h (Figure 6D).

Without the LSEC layer, the inflammatory reaction was less pronounced (Figure 5E–F), indicating the substantial participation of LSEC in the establishment of liver immune responses [22]. Moreover, the significant differences observed suggest that in the 3D-liver model, LSEC contribute to cytokine amounts either by a directional release into the underlying matrix or by modulating release from hepatocytes (Figure 5E–F for scheme). The data demonstrate that the hepatic cells in the 3D-liver model create a pro-inflammatory environment in response to the presence of *E. histolytica* (Figure 5E–F) and thus initiate an innate immune response regulated in time and space.

### Galectins as adhesion molecules for *E. histolytica* during 3D-liver model invasion

Galectin-1 and -3 exist in the extracellular milieu and in association with the surface of different cell types. Galectin-1 is characteristic of endothelial cells and galectin-3 is mainly present in epithelial cells [20]. The drastic reduction of amoebic invasion by the presence of galactose (Figure 3) prompted us to investigate the potential role of galectin-1 and -3 in *E. histolytica* adhesion to the human cells.

**Figure 7:**
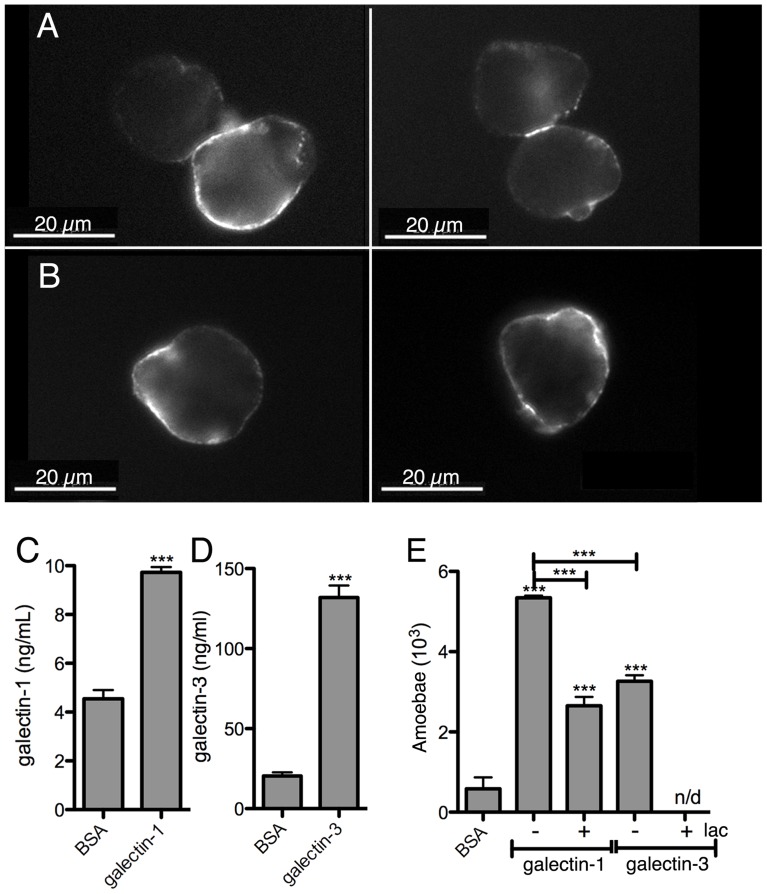
*E. histolytica* binding to human galectin-1 and -3. Bacterially expressed purified human galectin-1 and -3, and BSA as a control, were used for the incubations with the amoebae. Galectin-1 (A) or -3 (B) binding to the trophozoite surface, visualized by immunofluorescence with human galectin-specific antibodies. Quantification of galectin-1 (C) or -3 (D) binding to immobilized trophozoites by ELISA-like assays. (E) Trophozoite adhesion to immobilized galectin-1 or -3 and competition with lactose (lac).

The ability of *E. histolytica* to bind human galectin-1 and -3 was examined using bacterially expressed human recombinant proteins. Their binding to trophozoite surfaces after 25 min of incubation was observed in immunofluorescence experiments using anti-galectin antibodies. The signal intensity was variable in the amoebic population and the labelling was frequently unevenly distributed (Figure 7A and B), suggesting protein clustering. Binding was quantified using an ELISA-like assay (Figure 7C and D). Moreover, trophozoites adhere to surfaces covered with galectin-1 or -3 (Figure 7E).

**Figure 8:**
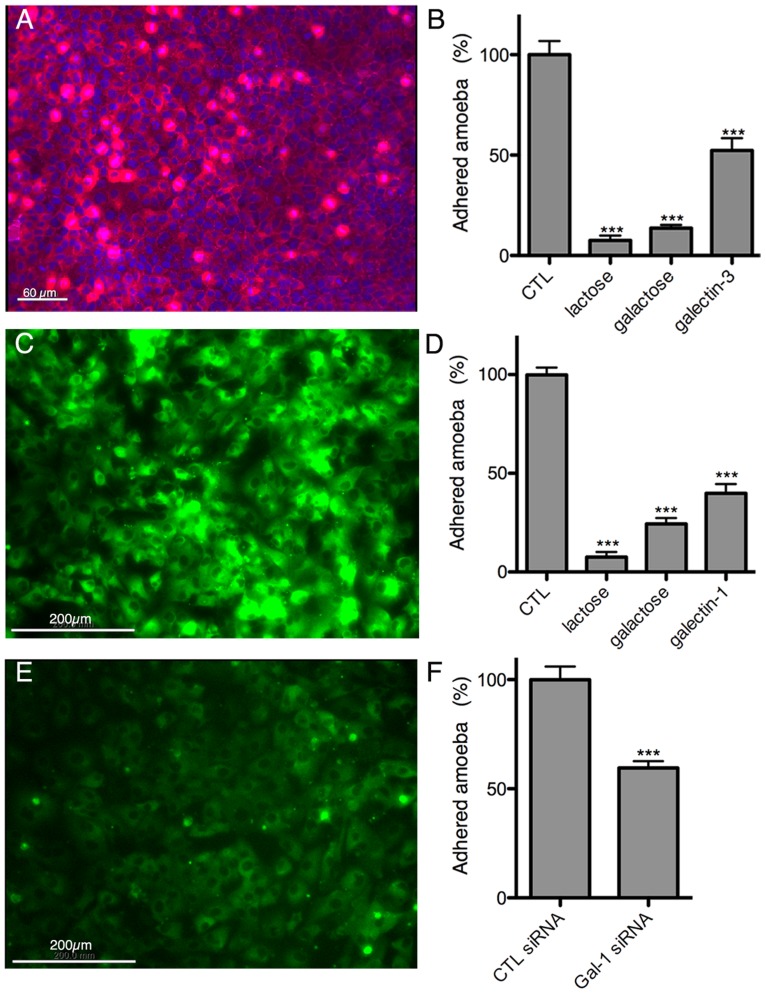
Galectin-1 and -3 dependent adhesion of *E. histolytica* to human hepatic cells. Immunofluorescence localization of cell surface-associated (A) galectin-3 on Huh-7 and (C) galectin-1 on LSEC. Amoeba adhesion to (B) Huh-7 or (D) LSEC 2D cultures Control (CTL), corresponding to incubations without proteins or sugars added, was set to 100%. (E) LSEC galectin-1 surface label 72 h after transfection with galectin-1 specific siRNA. (F) Amoeba binding to LSEC transfected with galectin-1 specific or unrelated control siRNA. Incubations and quantification of trophozoite binding as for B and D. Graphs with standard deviation and statistical evaluation by One way ANOVA, p<0.0001*** for 3 independent experiments.

Immunofluorescence experiments with the 3D-liver model revealed the presence of galectin-1 at the LSEC and of galectin-3 at the Huh-7 surface (Figure 8A,C). To test *E. histolytica* binding to the cell surface-associated galectins of the hepatic cells, Huh-7 (Figure 8B) and LSEC (Figure 8D) monolayers were incubated with the trophozoites in the presence or the absence of either the recombinant galectins, or galactose and lactose, both sugars known to inhibit human galectins (lactose stronger than galactose) and amoebic lectins as Gal/GalNac (galactose stronger than lactose), and the number of amoebae adhered to the human cells was determined. Trophozoite adhesion was drastically diminished by the sugars or the recombinant proteins (Figure 8B and D), indicating for the first time the ability of *E. histolytica* to bind and adhere to human galectin-1 and -3.

To further analyse the dependence of amoebic adhesion upon human surface galectin, we focussed on LSEC as the first target of amoeba interaction and performed siRNA knock-down experiments for galectin-1. Galectin-1 specific siRNA strongly reduced the level of surface galectin-1 in transfected LSEC (Figure 8E) and trophozoite adhesion was decreased by around 40% (Figure 8F), demonstrating the participation of surface-associated galectin-1 in *E. histolytica* adhesion to LSEC.

### Stimulation of the hepatic cell inflammatory response by galectin-1 and -3

**Figure 9:**
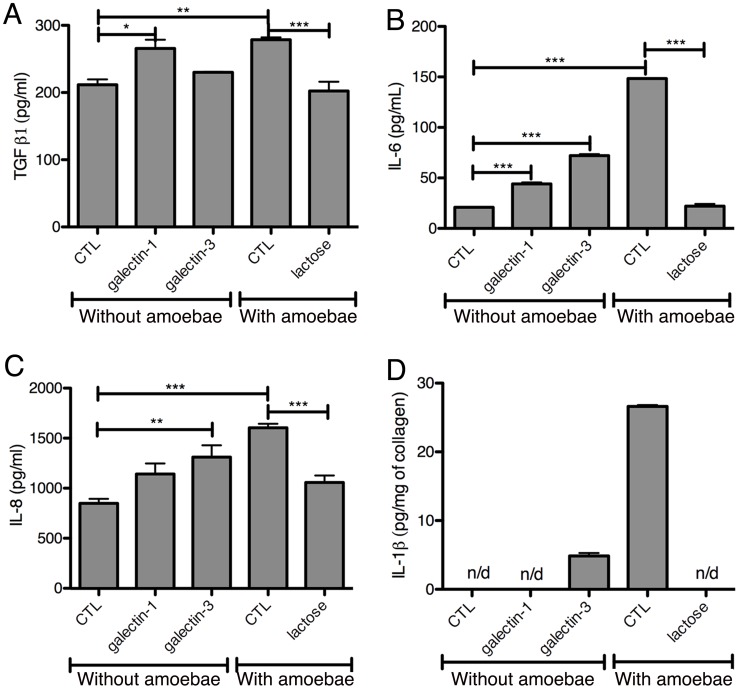
Induction of a pro-inflammatory response by galectin-1 and -3 or *E. histolytica*. Cytokine quantification by ELISA assays in fractions S1 (A–C) or S3 (D) after 6 h of incubation of 3D-liver models with bacterially expressed purified human galectin-1 or -3 (1 µg/ml) or *E. histolytica*. Control (CTL) corresponds to no protein added. Incubations with amoebae were performed in the presence of lactose or in its absence (CTL). Graphs with standard deviation, statistical evaluation by One way ANOVA, p<0.05*, p<0.001**, p<0.0001*** for (A–C) and values significantly different from 0 for galectin-3 p = 0.0068, CTL p = 0,0049 (D), for 3 independent experiments. n/d = not detected.

The potential immuno-modulatory role of galectin-1 and -3 in the 3D-liver model was examined by testing the ability of the recombinant proteins to modify cytokine release (Figure 9A–D). Bacterially expressed galectin-1 and -3 recombinant proteins (1 µg/ml) were added for 6 h to the medium on top of the 3D-liver model in the absence of amoebae and cytokine amounts quantified by ELISA (Figure 9A–D). Galectin-3 promoted the release of IL-1β and significantly increased IL-6 and IL-8 levels. Galectin-1 promoted the IL-6 and TGF-β1 release. In addition, lactose competition in the presence of *E. histolytica* (Figure 9) completely abolished the enhanced cytokine release observed. To exclude that the stimulation was induced by bacterial endotoxin contaminations, we performed a control using galectin-1 purified from human cell lines (note that galectin-3 was not available) and obtained a similar capacity to induce the cytokine release.

Overall the data suggest an important immuno-regulatory role for galectin-1 and -3 through the stimulation of the release of pro-inflammatory cytokines, which may be relevant for the induction of the host response during liver invasion by *E. histolytica*.

## Discussion

Liver abscesses are a fatal feature of infection with *E. histolytica*. Host and parasite factors leading to liver infection remain largely unknown. An important question is how this parasite adheres to and crosses the liver endothelium. Based on our previous data, we hypothesized in this work that amoebic Gal/GalNAc lectin and surface-bound (or secreted) human factors are involved in this key step of liver invasion. However, the existing experimental systems (standard cell culture and animals) did not allow the molecular analyses necessary to test this hypothesis.

We thus established a new human 3D-liver model, reproducing main characteristics of hepatic sinusoids, designed for the study of *E. histolytica* infection. It is the first 3D-liver model using cells of human cell lines co-cultured in a 3D COL-I scaffold in the sandwich approach. The latter was chosen to obtain a hepatic sinusoid-like organization of the cells [12] [23] in a 3D architecture, which is more physiologically relevant than strategies like 2D systems [9] or 3D cellular spheroids [11]. Though our 3D-liver model was built with only a single collagen type, the hepatic cells remodelled the matrix and released further ECM components and adhesive proteins, suggesting that the cells are able to diversify the initially homogenous COL-I matrix. We showed that hepatocytes maintain several physiological functions beyond the time-point used for our analyses and LSEC express the surface receptors ICAM-1 and integrin-β1, and exhibit barrier function. Major advantages of this 3D-liver model are: it is human-relevant (uses human cells); preserves a physiological context (mimics the hepatic sinusoid architecture), presents a controlled, reproducible *in vitro* environment.

One appealing perspective is the use of the 3D-liver model for the study of other important parasitic or viral hepatic infections, but specific adaptations to each of the pathogens under investigation will be required. Notably, the inclusion of other liver-resident (stellate and Kupffer cells) and immune (monocytes, macrophages, NKT cells) cell types, blood components related to the innate immune response, variations in the oxygen concentration or the application of flow to mimic mechanical forces of the blood stream can be envisaged.

**Figure 10:**
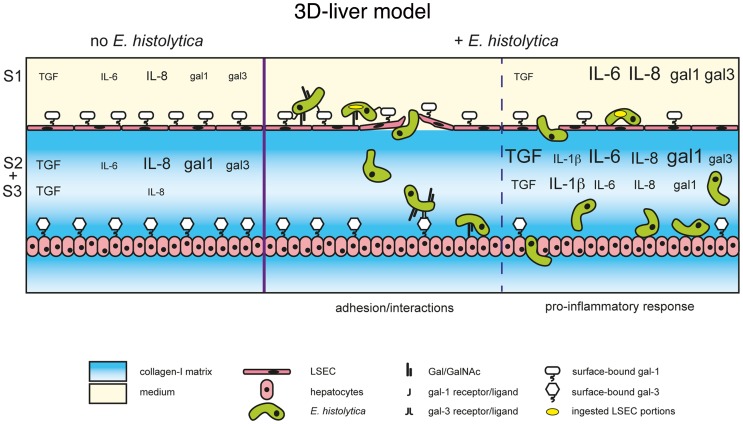
The human 3D-liver model, the dual role of galectin-1 and -3 during *E. histolytica* invasion. Schematic presentation of the *E. histolytica* invasion mechanisms at the 3D-liver model: the early LSEC adhesion interactions and crossing as well as the induction of the pro-inflammatory cytokine release into the different compartments of the 3D-liver model. The summary highlights the involvement of amoebic Gal/GalNAc lectin in the interactions with the human cells, and the dual role of human galectin-1 and 3. First, surface-associated galectins promotes amoeba adhesion during the early stages, and second, released galectins participate in the induction of the inflammatory response later in the invasion process.

The 3D-liver model here described was used to analyse the initial events occurring upon *E. histolytica* interactions with hepatic host cells. We examined human cell responses and parasite abilities to cross the endothelial barrier. We identified new key elements of amoeba-cell interactions triggering a pro-inflammatory response (the data are summarized in Figure 10). For the first time, we have discovered the role of amoebic Gal/GalNAc lectin in the amoebic crossing of the endothelial barrier and a function of human galectins in amoebic adhesion. Also for the first time, we were able to characterize the spatio-temporal distribution of the components of the pro-inflammatory response against *E. histolytica* in a hepatic human-relevant system.

The better characterization of the *E. histolytica* liver inflammatory process in the human host is an important issue for the understanding of the disease. In fact, neutrophils, macrophages and T cells have been related to the local host immune responses in human amoebic abscesses [24], but the human host inflammatory response during *E. histolytica* liver invasion is poorly known. Though it appears relatively controlled over time (i.e. restricted to areas surrounding amoebae-containing foci, mainly single abscess of restricted size in humans), it likely contributes to amoebic progression, diminished liver function, and clinical complications [25].

Here we showed that during amoebic invasion of the 3D-liver model several cytokines (IL-1β, IL-6, IL-8 as well as galectin-1) accumulated in the matrix-associated fraction. Although cytokine accumulation in ECM has been documented and their retention proposed as an essential mechanism for the establishment of gradients and compartments [26], little is known on the functions of these ECM-associated molecules. Nonetheless, ECM-associated galectin-1 is able to trigger T cell death at lower concentrations than the soluble form [27]. In the context of liver infection, it will be interesting to examine the hypothesis that ECM-associated galectin-1 is able to trigger cell death.

We also demonstrated the binding of soluble galectin-1 and -3 to amoebae and the participation of native (i.e. cell surface-associated) galectin-1 and -3 in amoebic adhesion to LSEC and hepatocytes. Moreover, incubation of the 3D-liver model with recombinant galectin-1 or -3 induced an inflammatory response, similar to the response to the presence of amoebae, though less complete. From all these data we can suggest a dual role of galectin-1 and -3 during amoebic hepatic infection. First, cell surface-linked galectin-1 and -3 promote amoebic adhesion to human liver cells, and second, released galectin-1 and -3 induce a pro-inflammatory hepatic response. It is possible that parasite binding to human cells directly facilitates the galectin release, but more data will be necessary to conclude on this.

Amoebic liver abscess formation based on carbohydrate-sensitive adherence mechanisms is here for the first time suggested at the molecular level, by the discovery of the simultaneous participation of both parasite and human lectins in the invasion of the 3D-liver model. We do not know if amoebic Gal/GalNAc and human galectins interact in a direct way through sugar residues. In fact, galectin-1 and -3 are released into the extracellular milieu by a non-classical secretion pathway and thus seem not to be glycosylated in the ER-Golgi trafficking pathway [28]. Here we found that bacterially expressed galectins (i.e. not glycosylated) bind to *E. histolytica* and that binding was blocked by carbohydrates (lactose), but we did not formally demonstrate that this binding depends on the lectin function of human galectins.

Galectin-1 and -3 are the most ubiquitously expressed and extensively studied members of the galectin family. Several immuno-regulatory functions have been discovered for galectin-1 and -3 in acute and chronic inflammation [29]. For instance and of relevance for our findings, up-regulated galectin-3 expression during *Toxoplasma gondii* hepatic infection seams to exert an important role in innate immunity, including a pro-inflammatory and a dendritic cell regulatory effect [19]. We conclude that both, the adhesive and the immuno-regulatory role of galectin-1 and -3 we detected are relevant for the induction of the host response during liver invasion by *E. histolytica*.

## Materials and Methods

### Human 3D-liver model

A 3D COL-I matrix was polymerized in 35 mm diameter cell culture dishes (ibidi 81156) by adding 650 µl of a bovine COL-I (Nutagen, Advanced Biomatrix) solution (1.0 mg/ml) in DMEM (31966-047 Invitrogen) and incubating for up to 1 h at 37°C in a humidified incubator at 5% CO_2_. A suspension of hepatocytes (4×10^5^) from the human Huh-7 cell line (JCRB0403, JCRB cell bank) was added on top of the polymerized matrix and incubated overnight (37°C, 5% CO_2_) in complete DMEM medium (i.e. with 10% foetal bovine serum). The medium was replaced by 150 µl of the same COL-I solution. After polymerization (40 min, 37°C) of the new COL-I matrix layer, cells (3×10^5^) from the LSEC line (Salmon et al., 2000) were plated on the top and the co-cultures incubated for 48 h (37°C, 5% CO_2_) in complete DMEM with daily medium change. The setups consisting of hepatocytes (4×10^5^) embedded in the 2 COL-I matrix layers without LSEC or of an LSEC layer (4×10^5^) on the top of the COL-I sandwich without hepatocytes were prepared identically. Note that the cell densities seeded were chosen to obtain monolayers for both cell types. Two-photon microscopy (multiphoton microscope LSM710_NLO upright) was used for visualization and acquisition of 3D images. Hepatic cells were labelled with 2.5 µM red cell tracker (CMTPX, Invitrogen C34552; 30 min pre-incubation) and the COL-I matrix fibres were detected by the second harmonic generation (SHG) signal (Chameleon laser, λ = 800 nm). To decrease background fluorescence the samples were incubated 12 h prior to analysis with complete DMEM medium without phenol red (Invitrogen 31053) with NEAA (GIBCO 1140-035), 4 mM glutamine (GIBCO 25030-024) and 1 mM sodium pyruvate (GIBCO 11360-039) added and images acquired in the same medium without serum.

### COL-I matrix porosity

COL-I matrix fibres were visualized by two-photon microscopy using SHG. 3D images were subjected to thresholding to convert matrix pores to white and collagen fibrils to black objects. The porosity of each focal plane (1 µm) of each image was evaluated using the “analyze particle function” of ImagePro software with the black fibres being defined as the particles. A total of 3 images of 3 independent experiments were analysed.

### Real-time quantitative PCR

3D-liver models, the setup without LSEC, and Huh-7 cell standard 2D cultures, cultivated for 3–14d, were independently transferred to Eppendorf tubes and centrifuged for 5 min at 16000 g. The pellets (containing cells and the COL-I matrix) were lysed with lysis buffer from the RNAeasy Plus Mini kit (Qiagen) and total RNA was prepared with the kit. Reverse transcription was performed with Superscript II enzyme (Invitrogen-18064-022) and 3 µg RNA. Reactions without reverse transcriptase added were used as controls. Real-time quantitative PCR amplification was carried out using Power SYBR Green PCR Master Mix (Perkin Elmer Applied Biosystems) and 2 µM of the primers and a Quantstudio 7 Flex QPCR system (Applied biosystems, life technologies). Human genes and primers used are listed in Text S1. Results were normalized to human GAPDH transcript levels. Changes in expression levels over time of culture were expressed relative to the levels determined for the corresponding 3d culture. The differences between the culture conditions at a given timepoint were expressed relative to the levels observed with 2D Huh-7 cultures.

### Permeability of the human 3D-liver model

Permeability of LSEC monolayers or the COL-I matrix was determined through the ability of 1 µm diameter fluorescent carboxylate microspheres (Polysciences, 15702) to pass through the cell layer or the matrix border. After 3 h of incubation, the beads added on top of the samples, the COL-I matrix and the cells were visualized by two-photon microscopy. For each microscopic field (0.28 mm^2^) the x-, y- and z-position of each fluorescent bead and the z-position of the cell layers or the upper matrix border were determined using ICY software (http://icy.bioimageanalysis.org). A LabMat script was created to represents the z-stack position of individual beads, with the matrix border or the LSEC layer position set to 0 µm.

### Immunofluorescence, cellular markers and confocal microscopy analysis

3D-liver models were fixed with 4% formaldehyde for 30 min. Blocking and incubation with the first antibody was (overnight in PBS containing 1% BSA) was followed by 2 h of incubation with secondary antibodies (Cell Signalling) in the same buffer. Immunostaining was performed with antibodies specific for human integrin-β1 (Millipore, 3199Z), ICAM-1 (R&D systems, 11C81) and E-cadherin (Cell Signaling, 24E10). Images were acquired with a multiphoton LSM710_NLO upright microscope, using the multi-photon laser and SHG to visualize the COL-I matrix, or the confocal laser the reflection mode to visualize the COL-I matrix and cell topography.

To determine the intensity of E-cadherin labelling, 1 µm diameter fluorescent carboxylate microspheres (Polysciences, 15702) were used as reference. Fluorescence intensities of E-cadherin signals and the beads was measured using Zen software from Zeiss. The intensity of the E-cadherin label was normalized by the intensity of the beads (added prior to microscopy) present in the same field and focal plane (set as 100%).

### *E. histolytica* culture and invasion assay

Trophozoites of the *E. histolytica* strain HM1:IMSS were cultivated in TY-S-33 medium as described [30]. The virulence-attenuated HM1:IMSS strain is a long-term *in vitro* cultivated (i.e. more than 10 years in axenic culture) derivative, having lost the ability to induce liver abscesses in the hamster, while maintaining adhesive and cytotoxic activities when tested with standard 2D mammalian cell cultures [31]. Strains modified for the Gal/GalNAc lectin activities CTL and HGL-2 were previously published [4]. For invasion experiments, amoebae were collected from exponentially growing cultures and labelled with 2.5 µM green cell tracker (CMFDA, Invitrogen C2925) for 1 h. Labelled trophozoites (6×10^4^) were added in 2 ml serum-free DMEM without phenol red to the top of the model, the setup without LSEC or Huh-7 or the COL-I matrix without cells. After 1.5 h or 3 h of incubation, invasion was evaluated by two-photon microscopy and SHG. For each microscopic field (0.28 mm^2^) the x-, y- and z-position of each amoeba and the z-position of the cell layers or the upper matrix border were determined using ICY software. The rate by which amoebae cross the LSEC layer or the matrix was calculated by the quantification of the number of amoebae having a z-position bigger than the z-position of the LSEC or the matrix border and the normalization with the total number of amoebae per field. Data were represented as the percentage of amoebae able to cross. The invasion rate (i.e. migration towards hepatocytes) was obtained by considering the amoeba position relative to the position of the LSEC and the hepatocyte layer set as 0% and 100%, respectively. For each condition, 10 fields were examined, in 5 independent experiments.

### Growth factor and cytokine quantification

Samples were prepared from 3 compartments of the 3D-liver model: S1 (medium on top of, i.e. outside), S2 (medium inside) and S3 (extracted from the non-soluble fraction containing cells, matrix and matrix-associated molecules). After collecting the S1 fraction, the remaining material was transferred to an Eppendorf tube and centrifuged for 5 min at 16000 g. The supernatant corresponds to the S2 fraction. The pellet containing the non-soluble components was resuspended in DMEM and incubated with collagenase (2.5 mg/ml) for 15 min at 37°C to cleave the COL-I fibres. After centrifugation, the supernatant was used as fraction S3. Cytokines and growth factors were quantified by ELISA assays (R&D systems) for human IL-1β, IL-6, IL-8, TGF-β, TNF-α, IFN-γ, HGF, acidic FGF, VEGF, galectin-1 and -3.

### Sample preparation for proteomic analysis

To identify compounds released by the hepatic cells into the culture medium on top of the 3D-liver model, fresh serum-free DMEM was added and collected after 3 h. A total of 15 samples (Table S1 with complete raw data) was collected: 2 biological replicates of the control COL-I matrix without hepatic cells (1A and B); 4 biological replicates of the 3D-liver model in the absence (2A–D) and 9 biological replicates in the presence of *E. histolytica* (3A–I). Proteins contained in the collected medium were precipitated with methanol-chloroform [32] redissolved in 8M urea in 25 mM NH_4_HCO_3_, reduced with 5 mM TCEP (45 min, 37°C), alkylated with 50 mM iodoacetamide (60 min, 37°C) in the dark, diluted to 1 M urea and digested for 16 h at 37°C with trypsin (1 µg, sequencing grade gold, Promega). Peptide mixtures were acidified to pH 2.8 with formic acid, desalted with minispin C18 columns (Nest Group), vacuum-dried and solubilized in 0.1% formic acid and 2% acetonitrile for mass spectrometry. Liquid chromatography–mass spectrometry (LC-MS/MS) analysis, proteome data processing and analysis methods are described in Text S1.

### Galectin binding assay

Trophozoites (2×10^5^) were incubated for 25 min at 4°C in serum-free DMEM containing 5 µg/ml of bacterially expressed recombinant human galectin-1 or -3 (Prepotech; guaranteed for endotoxin levels less than 0.1 ng/µg), human galectin-1 expressed in human cell lines (Creative BioMat LGALS1) or BSA as a control. Amoebae were washed twice with serum-free medium, fixed with 4% formaldehyde, harvested, seeded (6×10^4^ per well) into 96-well Immunoplates (Nunc) and prepared for immunofluorescence staining. Immunofluorescence labelling was performed with anti-galectin-1 or -3 antibodies (Cell Signalling, D6008T and abcam, 31707) and secondary antibodies coupled to AlexaFluor 555 (Cell Signaling). Images were acquired with a fluorescence microscope (Olympus IX81). For quantitative ELISA-like assays, trophozoites were harvested, allowed to adhere to wells of a 96 well plate (6×10^4^/well), incubated for 25 min with recombinant galectin-1, galectin-3 or BSA, gently washed and then incubated with biotinylated anti-galectin-1 or -3 antibodies (R&D systems). Colorimetric detection of antibody binding was obtained with streptavidin-HRP (R&D systems) and peroxidase substrate (SureBlue, KPL). Galectin-1 or -3 amounts in the samples were estimated using a standard curve established with the recombinant proteins.

### Adhesion to immobilized galectin-1 or -3

Wells of 96-well ImmunePlates (Nunc) were covered with recombinant galectin-1, -3 or BSA (10 µg/ml) (overnight at room temperature). Trophozoites labelled with green cell tracker and resuspended in 200 µl of DMEM without phenol red were added to the wells (6×10^4^ per well) and incubated for 30 min at 37°C in an anaerobic bag (Genbag Biomerieux, 45534). Wells were washed 3 times with serum-free DMEM. The number of adherent amoebae was quantified using fluorimetric detection of the cell tracker and normalized with a standard curve of known amoeba numbers.

### 2D hepatic cell adhesion assay

LSEC or Huh-7 monolayers grown for 72 h on COL-I coated (50 µg per ml) coverslips (i.e. conventional 2D culture conditions) were incubated for 30 min with the cell tracker-labelled trophozoites in the presence or absence of galactose (100 mM), lactose (100 mM) or recombinant galectin-1 or -3 proteins (10 µg/ml). Samples were washed 3 times and fixed with 4% formaldehyde. Fluorescent amoebae remaining adhered to the human cells were visualized by confocal microscopy and quantified using ICY software.

### LSEC galectin-1 silencing assay

LSEC monolayers grown overnight on COL-I coated ibidi dishes were transfected with 10 nM siRNA specific for galectin-1 (Santa Cruz, sc-35441) or negative control siRNA (Santa Cruz sc-37007 and Invitrogen, 4390843) using Lipofectamine RNAiMAX (Invitrogen, 13778) in Opti-MEM reduced serum medium (Invitrogen, 31985). After overnight incubation with the transfection solution, medium was changed and the dishes incubated for further 48 h. LSEC presented a significant reduction of the surface galectin-1 labelling at 72 h after transfection (evaluated by immunofluorescence intensity measurements, as described above). Amoebic adhesion was thus determined (as detailed above) at the 72 h time-point. Fluorescein-conjugated control siRNA (Santa Cruz sc-44239) was used to evaluate the transfection efficiency by fluorescence microscopy. After overnight incubation with the transfection solution, most of the LSEC were fluorescein-positive.

## Supporting Information

Figure S1 **Immunolocalisation of surface markers of LSEC and Huh-7 cells in the human 3D-liver model.** Immunofluorescence staining of 3D-liver models (after 3d of culture) with human-specific anti-integrin-β1 (A–C), -ICAM1 (D–E) and -E-cadherin (F) antibodies. Images were acquired with a multiphoton microscope using either confocal laser (A–C), visualizing the COL-I matrix and cell topography by the reflection mode (C, in green, superposed to the anti-integrin-β1 labelling in red), or multi-photon laser (D–F) visualizing the COL-I matrix with second harmonic generation signals (E, in blue, superposed to the anti-ICAM1 labelling in red). 3D reconstructed images at the LSEC (A and D) or transversal cuts at the hepatocyte layer z-position (B and C, E and F) using ICY software.
(TIF)

Figure S2 **LSEC monolayer barrier function in the 3D-liver model.** Permeability of LSEC monolayers or the COL-I matrix was analysed through the ability of 1 µm diameter fluorescent carboxylate microspheres to pass through the cell layer or the matrix border. See Figure 1 and 2 for a schematic drawing of the different setups. After 3 h of incubation the beads added on top of the samples and the components of the setups were visualized by two-photon microscopy. From 3 independent experiments, 10 microscopic fields (0.28 mm^2^) were analysed for each sample using ICY software. The graph represents the z-stack position of individual beads, with the matrix border or the LSEC layer position set to 0 µm. Red bars indicate the mean.
(TIF)

Figure S3 **Expression of hepatocyte functions in the human 3D-liver model.** Q-RT-PCR analysis for the expression of hepatocyte markers in standard 2D Huh-7 monocultures (2D), the COL-I sandwich with only a Huh-7 monolayer (no LSEC) and the 3D-liver model with a Huh-7 and LSEC layer (model). (A) Transcript amounts monitored over time of culture (3–14d) for 2D Huh-7 (in blue), no LSEC (in orange) and 3D-liver model (in magenta). Changes in transcript levels were expressed as log2-fold changes in comparison to levels determined for the corresponding 3d cultures (set to 0). (B) Transcript levels in 3d cultures represented as log2-fold changes over levels in 2D Huh-7 cultures (set to 0). Note that for the 3D-liver model, normalization of Q-PCR data with GAPDH (expressed by both hepatocytes and LSEC) leads to an underestimation of the transcript level for the hepatocyte-specific markers.
(TIF)

Figure S4 **E-cadherin labelling of Huh-7 cells during** ***E. histolytica*** **invasion of the 3D-liver model.** Immunostaining of 3D-liver models with human-specific anti-E-cadherin antibodies. Images were acquired with the multiphoton microscope and show E-cadhering labelling at the Huh-7 level without (A) and with (B) amoeba interaction for 6 h, in transversal cuts at the hepatocytes z-position (ICY software). Fluorescence intensity was measured using Zen software from Zeiss. The graph (C) represents the E-cadherin fluorescence intensity normalized by the fluorescence of 1 µm diameter fluorescent carboxylate microspheres in the same field and focal plane as 100% fluorescence intensity.
(TIF)

Table S1 **Human proteins identified in the raw MS data files by search with the** ***H. sapiens*** **UniProt database.** Each of the 15 samples (collagen-I controls, 3D-liver models in the absence and in the presence of *E. histolytica*) was analysed separately. The table contains data on the peptides found by LC-MS/MS, the accession numbers and descriptions of the corresponding proteins.
(XLSX)

Table S2 **Human proteins in the secretome of the 3D-liver model in the absence or in the presence of** ***E. histolytica.*** Human proteins were identified with the *H. sapiens* UniProt database and matches with the *B. taurus* UniProt database were removed.
(XLSX)

Text S1 **The file contains the list of primers used for the Q-PCR analysis, the Gene ID numbers from the LocusLink public database for the genes used in the Q-PCR analysis, the protein accession numbers from the UniProt database for the proteins given in** **Tables 1** **and** **2** **, as well as the methods for Liquid Chromatography–Mass Spectrometry (LC-MS/MS) analysis and proteome data processing and analysis.**
(PDF)
