# Supplementary figures and images for: A New Human 3D-Liver Model Unravels the Role of Galectins in Liver Infection by the Parasite Entamoeba histolytica
Source: PLoS Pathog. 2014 Sep 11;10(9):e1004381. doi: 10.1371/journal.ppat.1004381 (PMC4161482; doi:10.1371/journal.ppat.1004381)

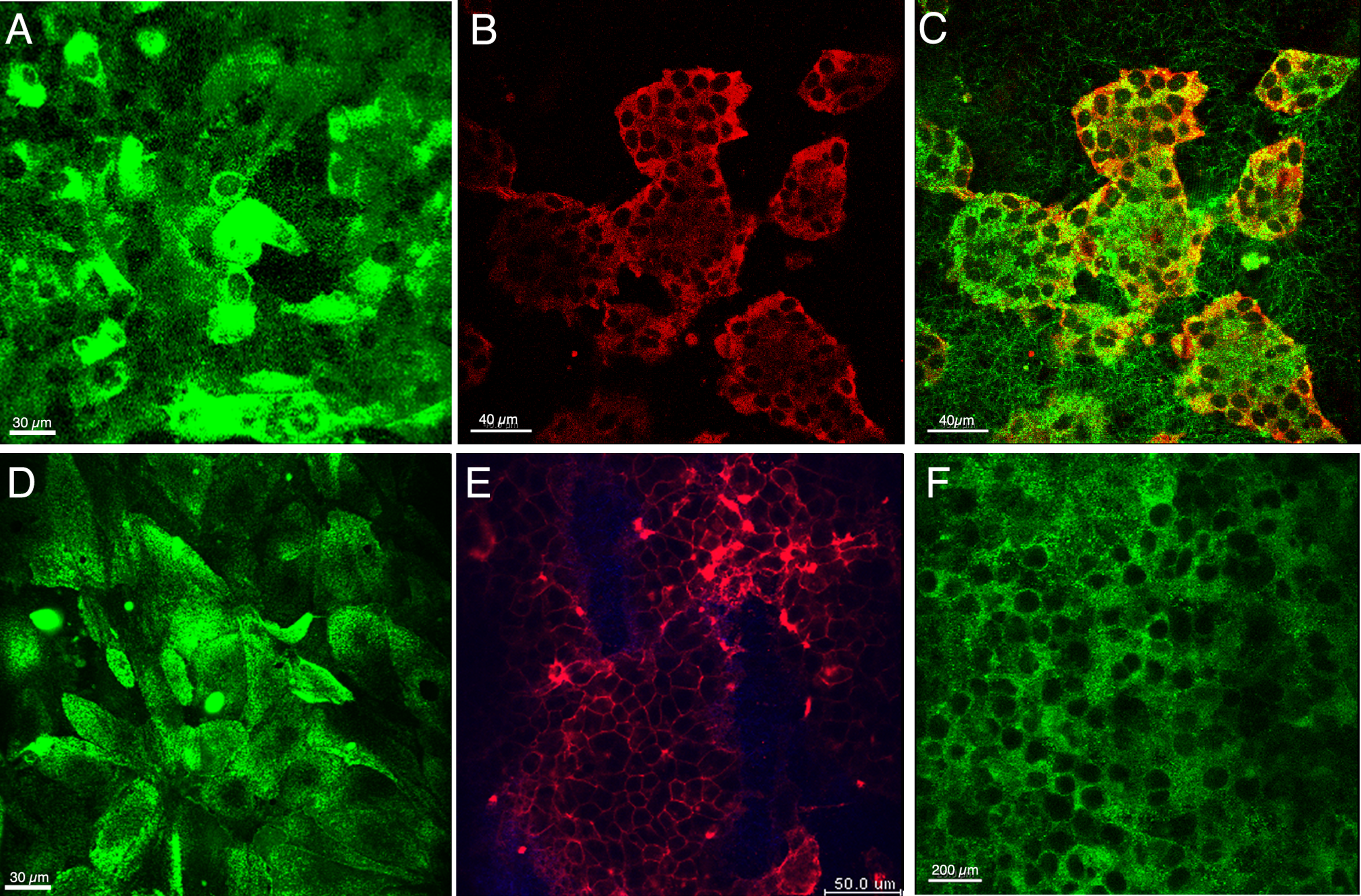

Supplement: Figure S1 — Immunolocalisation of surface markers of LSEC and Huh-7 cells in the human 3D-liver model. Immunofluorescence staining of 3D-liver models (after 3d of culture) with human-specific anti-integrin-β1 (A–C), -ICAM1 (D–E) and -E-cadherin (F) antibodies. Images were acquired with a multiphoton microscope using either confocal laser (A–C), visualizing the COL-I matrix and cell topography by the reflection mode (C, in green, superposed to the anti-integrin-β1 labelling in red), or multi-photon laser (D–F) visualizing the COL-I matrix with second harmonic generation signals (E, in blue, superposed to the anti-ICAM1 labelling in red). 3D reconstructed images at the LSEC (A and D) or transversal cuts at the hepatocyte layer z-position (B and C, E and F) using ICY software. (TIF) [file ppat.1004381.s001.tif]

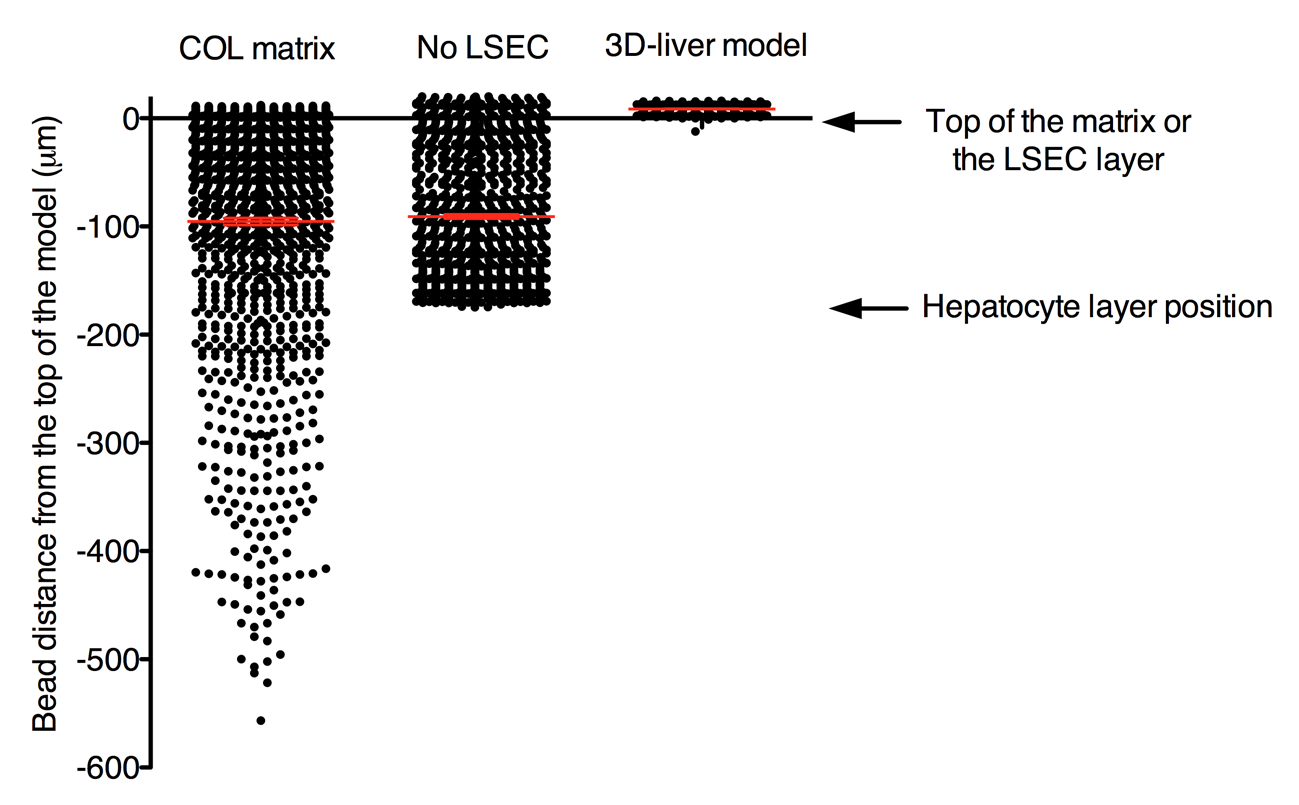

Supplement: Figure S2 — LSEC monolayer barrier function in the 3D-liver model. Permeability of LSEC monolayers or the COL-I matrix was analysed through the ability of 1 µm diameter fluorescent carboxylate microspheres to pass through the cell layer or the matrix border. See Figure 1 and 2 for a schematic drawing of the different setups. After 3 h of incubation the beads added on top of the samples and the components of the setups were visualized by two-photon microscopy. From 3 independent experiments, 10 microscopic fields (0.28 mm2) were analysed for each sample using ICY software. The graph represents the z-stack position of individual beads, with the matrix border or the LSEC layer position set to 0 µm. Red bars indicate the mean. (TIF) [file ppat.1004381.s002.tif]

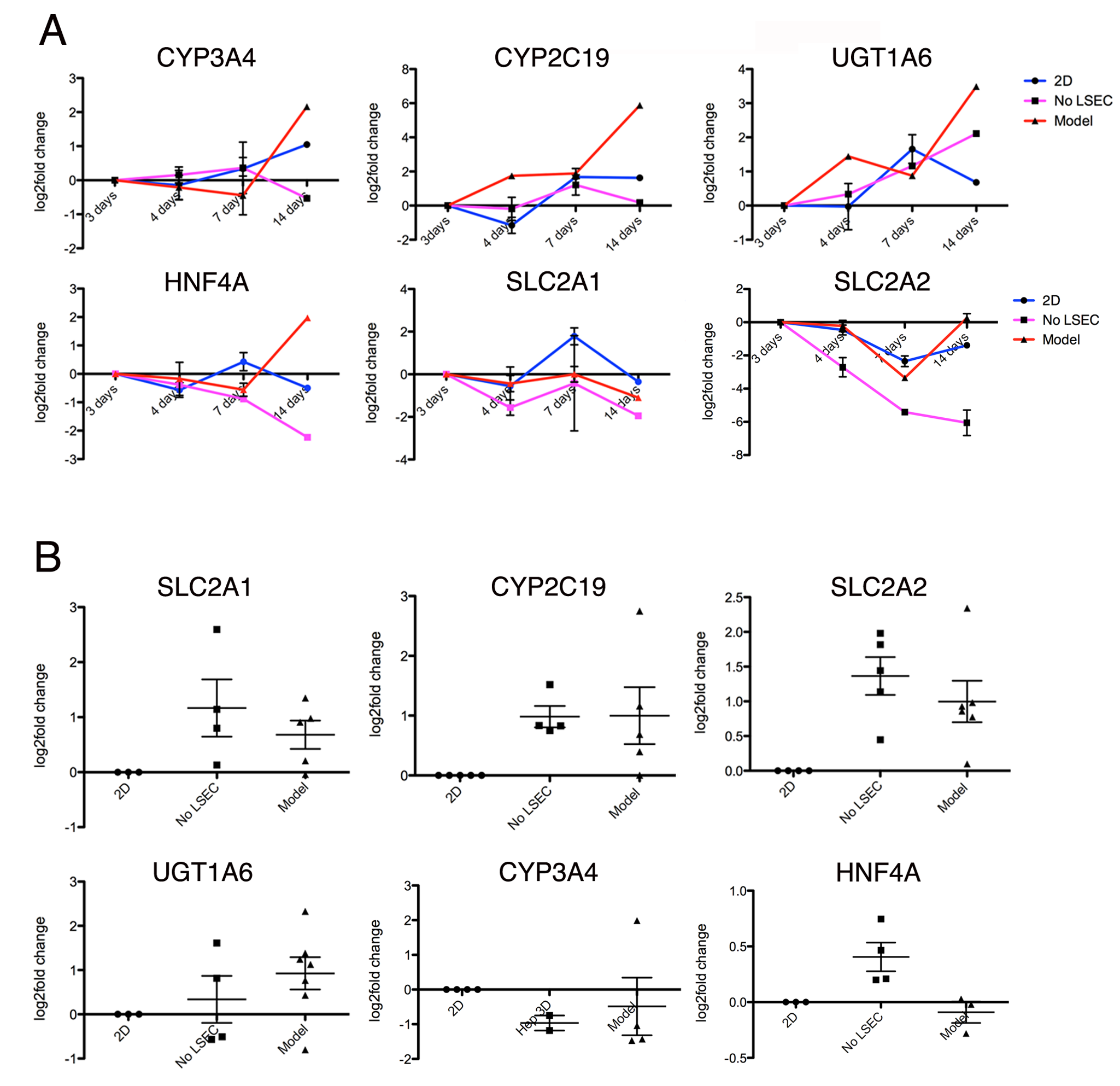

Supplement: Figure S3 — Expression of hepatocyte functions in the human 3D-liver model. Q-RT-PCR analysis for the expression of hepatocyte markers in standard 2D Huh-7 monocultures (2D), the COL-I sandwich with only a Huh-7 monolayer (no LSEC) and the 3D-liver model with a Huh-7 and LSEC layer (model). (A) Transcript amounts monitored over time of culture (3–14d) for 2D Huh-7 (in blue), no LSEC (in orange) and 3D-liver model (in magenta). Changes in transcript levels were expressed as log2-fold changes in comparison to levels determined for the corresponding 3d cultures (set to 0). (B) Transcript levels in 3d cultures represented as log2-fold changes over levels in 2D Huh-7 cultures (set to 0). Note that for the 3D-liver model, normalization of Q-PCR data with GAPDH (expressed by both hepatocytes and LSEC) leads to an underestimation of the transcript level for the hepatocyte-specific markers. (TIF) [file ppat.1004381.s003.tif]

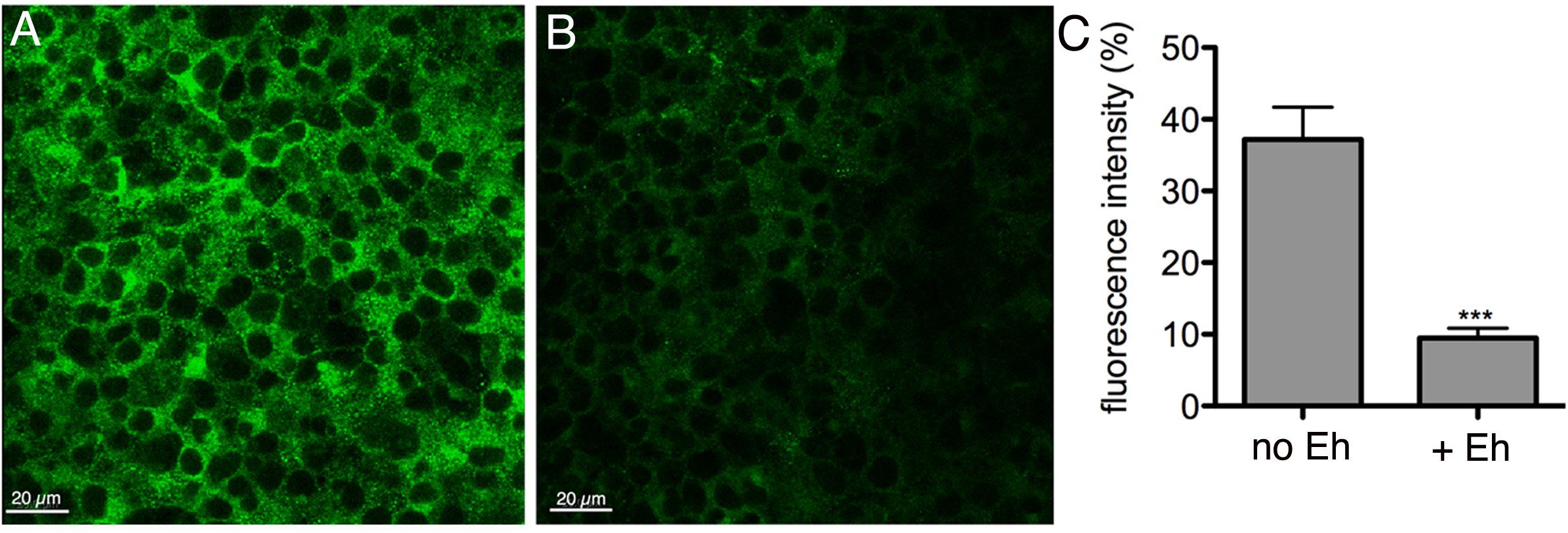

Supplement: Figure S4 — E-cadherin labelling of Huh-7 cells during E. histolytica invasion of the 3D-liver model. Immunostaining of 3D-liver models with human-specific anti-E-cadherin antibodies. Images were acquired with the multiphoton microscope and show E-cadhering labelling at the Huh-7 level without (A) and with (B) amoeba interaction for 6 h, in transversal cuts at the hepatocytes z-position (ICY software). Fluorescence intensity was measured using Zen software from Zeiss. The graph (C) represents the E-cadherin fluorescence intensity normalized by the fluorescence of 1 µm diameter fluorescent carboxylate microspheres in the same field and focal plane as 100% fluorescence intensity. (TIF) [file ppat.1004381.s004.tif]
