# Supplementary material for: A New Human 3D-Liver Model Unravels the Role of Galectins in Liver Infection by the Parasite Entamoeba histolytica
Source: PLoS Pathog. 2014 Sep 11;10(9):e1004381. doi: 10.1371/journal.ppat.1004381 (PMC4161482; doi:10.1371/journal.ppat.1004381)
Supplement: Text S1 — The file contains the list of primers used for the Q-PCR analysis, the Gene ID numbers from the LocusLink public database for the genes used in the Q-PCR analysis, the protein accession numbers from the UniProt database for the proteins given in Tables 1 and 2 , as well as the methods for Liquid Chromatography–Mass Spectrometry (LC-MS/MS) analysis and proteome data processing and analysis. (PDF) [file ppat.1004381.s007.pdf]

Text S1:

Supporting Information. A new 3D-liver model unravels the role of galectins in liver infection by the parasite *Entamoeba histolytica* by Petropolis et al.,

Primers used for the Q-PCR analysis

Cytochrome P450 2C19 (CYP2C19)
forward primer: GGAAAACGGATTTGTGTGGGA
reverse primer: GGTCCTTTGGGTCAATCAGAGA;
Cytochrome P450 3A4 (CYP3A4)
forward primer: AAGTCGCCTCGAAGATACACA;
reverse primer: AAGGAGAGAACACTGCTCGTG;
UDP Glucuronosyltransferase 1 Family, Polypeptide A6 (UGT1A6)
forward primer: TGGTGCCTGAAGTTAATTTGCT;
reverse primer: GGCTCTGGCAGTTGATGAAGTA;
Solute carrier family 2 member 1 (SLC2A1)
forward primer: GGCCAAGAGTGTGCTAAAGAA ;
reverse primer: ACAGCGTTGATGCCAGACAG ;
Solute carrier family 2 member 2 (SLC2A2)
forward primer GGGCAATTATGATCTGTGGCA;
reverse primer: TTCTGCTCACTCGATGCTTCT ;
Hepatocyte Nuclear Factor 4, Alpha (HNF4A)
forward primer : CGAAGGTCAAGCTATGAGGACA ;
reverse primer : ATCTGCGATGCTGGCAATCT ;
GAPDH
forward primer: TGCCAAATATGATGACATCAAGAA
reverse primer: GGAGTGGGTGTCGCTGTTG

Gene ID numbers from the LocusLink public database for genes used in the Q-PCR analysis

Cytochrome P450 2C19 [*Homo sapiens* (human)] (CYP2C19), gene number ID: 1557 ; Cytochrome P450 3A4 [*Homo sapiens* (human)] (CYP3A4), gene number ID: 1576 ; UDP Glucuronosyltransferase 1 Family, Polypeptide A6 [*Homo sapiens* (human)] (UGT1A6), gene number ID: 54578 ; Solute carrier family 2 member 1 [*Homo sapiens* (human)] (SLC2A1), gene number ID: 6513 ; Solute carrier family 2 member 2 [*Homo sapiens* (human)]

(SLC2A2), gene number ID : 6514 ; Hepatocyte Nuclear Factor 4, Alpha [*Homo sapiens* (human)] (HNF4A), gene number ID: 3172 ; glyceraldehyde-3-phosphate dehydrogenase [*Homo sapiens* (human)] (GAPDH), gene number ID: 2597

Protein accession numbers from the UniProt database for proteins of Tables 1 and 2

P02763-Alpha-1-acid glycoprotein 1 ; P08697-Alpha-2-antiplasmin, P02765-Alpha-2-HS-glycoprotein ; P02771-Alpha-fetoprotein ; P01019-Angiotensinogen ; P07355-Annexin A2 ; P08758-Annexin A5 ; P01008-Antithrombin-III ; P02647-Apolipoprotein A-I ; P02652-Apolipoprotein A-II ; P04114-Apolipoprotein B-100 ; P02649-Apolipoprotein E ; Q5FYB0-Arylsulfatase J ; P02749-Beta-2-glycoprotein 1 ; P61769-Beta-2-microglobulin ; P10909-Clusterin ; P02452-Collagen alpha-1(I) chain ; P02461-Collagen alpha-1(III) chain ; P00736-Complement C1r subcomponent ; P01024-Complement C3 ; P0C0L4 Complement C4-A ; P01031-Complement C5 ; P00751-Complement factor B ; P08603 Complement factor H ; P05156 Complement factor I ; D3XNU5 E-cadherin ; P02671 Fibrinogen alpha chain ; P02671 Fibrinogen alpha chain ; P02675 Fibrinogen beta chain ; P0267 Fibrinogen beta chain ; P02679 Fibrinogen gamma chain ; P02679 Fibrinogen gamma chain ; P02751 Fibronectin ; P09382 Galectin-1 ; P17931 Galectin-3 ; Q08380 Galectin-3-binding protein ; P06744 Glucose-6-phosphate isomerase ; P00738 Haptoglobin ; P02790 Hemopexin ; P05546 Heparin cofactor 2 ; P05546 Heparin cofactor 2 ; P51858 Hepatoma-derived growth factor ; P20036 HLA class II histocompatibility antigen, DP alpha 1 chain ; P05362 ICAM1 ; P05556 Integrin beta1 ; P19823 Inter-alpha-trypsin inhibitor heavy chain H2 ; P01042 Kininogen-1 ; P02750 Leucine-rich alpha-2-glycoprotein ; Q9ULH4 Leucine-rich repeat and fibronectin type-III domain-containing protein 2 ; P14174 Macrophage migration inhibitory factor ; P14543 Nidogen-1 ; P36955 Pigment epithelium-derived factor ; P05155 Plasma protease C1 inhibitor ; P05154 Plasma serine protease inhibitor ; P00747 Plasminogen ; P05121 Plasminogen activator inhibitor 1 ; Q8NBP7 Proprotein convertase subtilisin/kexin type 9 ; P02760 Protein AMBP ; Q5SWX8 Protein odr-4 homolog ; P01893 Putative HLA class I histocompatibility antigen, alpha chain H ; P78509 Reelin ; P02753 Retinol-binding protein 4 ; P02787 Serotransferrin ; P02768 Serum albumin ; O00391 Sulfhydryl oxidase 1 ; P05452 Tetranectin ;P10599 Thioredoxin ; P35442 Thrombospondin-2; Q15582 Transforming growth factor-beta-induced protein ig-h3 ; P00749 Urokinase-type plasminogen activator ; P02774 Vitamin D-binding protein ; P04004 Vitronectin

Liquid chromatography–mass spectrometry (LC-MS/MS) analysis

Samples (containing 1μg peptides) were separated by reverse-phase chromatography via Thermo Scientific Proxeon nano LC using a C18 picofrit analytical column (360μm OD, 75μm ID, 10μm tip, Magic C18 resin, 5*μ*m size, Newobjective, USA). The HPLC was coupled to an LTQ-Orbitrap Velos mass spectrometer (Thermo Fisher Scientific). Peptides were loaded onto the column and separated with a linear gradient of 5-40% of acetonitrile, 1% formic acid and at a flow rate of 300nL/min. Mass spectra were acquired in the LTQ Orbitrap velos with full MS scan (RP 30000) followed by 10 data-dependent MS/MS scans with detection of the fragment ions in the FTMS HCD mode (RP 7500). Target values were 1×10^6 for full FT-MS scans and 5×10^4 for FT-MS MS*n* scans. Ion selection threshold was set to 5000 counts.

Proteome data processing and analysis

Raw MS data files (Table S1) were processed with the Thermo Proteome Discoverer software suite (version 1.4) using SEQUEST search engine with peptide precursor mass and fragment ion mass tolerances set to 10ppm and 0.6Da, respectively. Carbamidomethylation on cysteine residues was used as fixed modification, and oxidation of methionine along with N-terminal acetylation as variable modifications. Raw spectra were queried against the *H. sapiens* and *E. histolytica* UniProt database with the false discovery rate (FDR) set to 0.01 for peptide and protein identifications using the percolator node. To reduce the complexity of comparative data analysis, proteome data were grouped into 3 datasets, i.e. COL-I control (2 samples, 1A and B), model without amoebae (4 samples, 2A-D) and model with amoebae (9 samples, 3A-I). These datasets were searched against the *Bos taurus* UniProt database. Peptides/proteins identified in the *H. sapiens* database search were compared to those identified with the *B. taurus database* search, to identify contaminations with bovine proteins. Matches were filtered out, resulting in a final list of human database specific peptide matches. The table of the 64 proteins identified for the 3D-liver model without amoebae was obtained by requiring proteins to be identified in any of the biological replicates. The table for the 3D-liver model with amoebae contained 139 proteins specifically found in presence of amoebae (Table-

secretome-summary.xlsx). The raw data files were submitted to ProteomeXchange Consortium (http://www.proteomexchange.org) via the pride partner repository with the dataset identifier PXD000893.
